
JOURNAL INFORMATION
==============================
NLM Title Abbreviation: Innov Surg Sci
Journal ID: Innov Surg Sci
NLM Journal ID: 101708165
Journal ID: iss

Innovative Surgical Sciences

EISSN: 2364-7485
Publisher: De Gruyter

ARTICLE INFORMATION
==============================
PMCID: PMC13068491
DOI: 10.1515/iss-2026-2004
Article ID: iss-2026-2004
Article version: 1
Subjects: Article

4. Surgical Research

Electronic publication date: 2026 Apr 10
Publication date: 2026 Apr
Volume: 11
Issue: Suppl1
First page: 330
Last page: 385
Copyright: © 2026 by Walter de Gruyter Berlin/Boston
Copyright year: 2026
Copyright holder: De Gruyter
License: This work is licensed under the Creative Commons Attribution 4.0 International License.
License URL: https://creativecommons.org/licenses/by/4.0/

Page count: 56

==============================

DE GRUYTER BRILL

**Biomechanical Evaluation of Fixation Methods for Porcine Intestine in Surgical Research**
ID: E-DCK-449

**Surgical Research**
**Biomaterials**

Saskia Janett[1], Carina Micheler[2 3], Andreas Obermeier[2], Rainer Burgkart[2], Philipp-Alexander Neumann[1], Kamacay Cira[1]

[1]Department of Surgery, Klinikum Rechts Der Isar, TUM School of Medicine and Health, Technical University of Munich, München, Deutschland, [2]Department of Orthopaedics and Sports Orthopaedics, Klinikum Rechts Der Isar, TUM School of Medicine and Health, Technical University of Munich, München, Deutschland, [3]Institute for Machine Tools and Industrial Management, TUM School of Engineering and Design, Technical University of Munich, München, Deutschland

**Background:**
Understanding biomechanical properties of intestinal tissue is essential for advancing knowledge in gastrointestinal anastomotic healing. Tensile strength testing is one of the most standardized and reliable approaches for *ex vivo* evaluation. However, fresh intestinal tissue is not always available and the use of fixation techniques may alter the biomechanical characteristics of the samples and affect the validity of test results. This study aims to investigate and compare the biomechanical impact of commonly used preservation techniques on porcine small intestine with that of fresh tissue.

**Material and methods:**
A Zwick/Roell tensile testing machine was used to apply longitudinal force to porcine intestinal segments. Two synchronized cameras documented the tearing process from different angles. We evaluated stress-strain behaviour, stiffness, and maximum breaking strength across three preservation methods: frozen-thawed, ethanol (35%), and formalin-fixed. Fresh intestine was incubated in PBS for same-day testing, served as the control. Each preservation method was assessed after 7 and 14 days. In total, 70 segments (n=10 per group) were analysed. Stress-strain curves were generated, and mechanical parameters were computed using a custom MATLAB algorithm.

**Results:**
Significant differences in stiffness were observed compared to fresh tissue for frozen-thawed ($p$ = 0.0238) and formalin-fixed ($p$ = 0.0036) samples, while ethanol-preserved tissue showed no significant change ($p$ = 0.2348). Duration of storage also affected outcomes: stiffness differed significantly after 14 days for frozen-thawed tissue ($p$ = 0.0248) and already after 7 days for formalin-fixed samples ($p$ = 0.0024).

**Conclusion:**
Both preservation technique and storage duration substantially influence the biomechanical properties of porcine intestine. These findings are crucial when designing reliable and reproducible *ex vivo* models in surgical research. Moreover, they highlight the broader importance of accounting for preservation-related changes in biomechanical studies involving any biological tissue. Further investigations should explore ways to reduce such alterations and enhance the comparability of preserved and fresh specimens.

Open Access. © 2026 The Author(s), published by De Gruyter. This work is licensed under the Creative Commons Attribution 4.0 International License.

DE GRUYTER BRILL

### Not all patients are ready for utilization of AI in healthcare: a cross-sectional prospective study

ID: E-DCK-327

**Surgical Research**
**Clinical Studies**

Athanasios Zamparas[1], Dimitrios Trompoukis[1], Nicola Beger[1], Gulnar Jafarova[1], Nikolaus Börner[1], Jens Werner[1], Bernhard Renz[1], Ughur Aghamaliyev[1]

[1]LMU Klinikum, Allgmein, Viszeral & Transplantationschirurgie, München, Deutschland

**Background:**
Artificial intelligence (AI) is increasingly embedded in perioperative workflows, yet adoption depends on patient acceptance for specific clinical tasks. Evidence comparing acceptance between patients with malignant and benign surgical indications is limited. We evaluated overall acceptance, task-level preferences, and whether diagnosis category (malignant vs benign) modifies acceptance.

**Material and methods:**
Prospective cross-sectional survey at a university tertiary center (visceral surgery wards and clinics). Adults scheduled for elective abdominal surgery completed a 27-item German Likert questionnaire spanning: (i) supportive AI (lab/diagnostic assistance; intra-operative monitor guidance; simulation training), (ii) autonomous AI (autonomous anamnesis/consent; AI-delivered diagnosis; fully robotic operation), (iii) tele-surgery, and (iv) data-sharing for individualized risk. Responses were coded 1–5 (positive→negative); “unsure/no opinion” was coded 6 and excluded from acceptance denominators. The primary endpoint compared pooled acceptance of supportive vs autonomous AI. Secondary endpoints assessed tele-surgery, data-sharing, and item-level acceptance. Prespecified subgroup analyses: malignant vs benign diagnosis (per protocol), age (<70 vs ≥70), and planned procedure complexity (0=small, 1=medium, 2=major). Multivariable logistic regression (outcome: high supportive acceptance, ≥50% supportive-item positives) adjusted for age, sex, higher education, privacy concern, diagnosis category, and complexity.

**Results:**
We analyzed 200 patients (median age 59 years; 87/200 women, 43.5%; 93/200 malignant, 46.5%). Pooled acceptance was 73.9% for supportive AI and 33.5% for autonomous AI (absolute difference +40.4 pp; p<0.001). Data-sharing for individualized risk was 63.3%. Item-level acceptance was highest for simulation training (87.1%) and intra-operative monitor guidance (82.4%) and lowest for robot-led informed consent (16.6%) and fully robotic operation (27.6%).
Diagnosis category (malignant vs benign). Across pooled domains (supportive, autonomous, tele-surgery) and key items (simulation, monitor guidance, robot-led consent, fully robotic operation, data-sharing), no statistically significant differences were detected between malignant and benign cohorts after multiple simple proportion tests; effect sizes were small and directionally inconsistent. In multivariable analysis, malignant diagnosis was not an independent predictor of high supportive acceptance (adjusted odds ratio [AOR] 2.40, 95% CI 0.51–11.27; p=0.268).
Planned procedure complexity. Patterns were consistent across small (n=84), medium (n=44), and major (n=72) procedures; cross-strata $\chi^2$ tests for supportive AI, autonomous AI, tele-surgery, and data-sharing were not significant (all p>0.19).

Age (≥70 vs <70). Supportive acceptance remained high in both strata (no significant difference), while tele-surgery acceptance was lower in ≥70 (two-proportion test p=0.0017). Willingness to undergo a fully robotic operation was numerically lower in ≥70 without statistical significance.
Determinants. Female sex was associated with lower odds of high supportive acceptance (AOR 0.36, 95% CI 0.15–0.82; p=0.016). Higher education trended toward higher odds (AOR 2.13, 95% CI 0.85–5.32; p=0.106). High privacy concern trended toward lower odds (AOR 0.45, 95% CI 0.18–1.14; p=0.091). Age ≥70 and procedure complexity were not significant.

**Conclusion:**
Patients clearly prefer surgeon-supervised, supportive AI that enhances diagnostic and intra-operative decision-making, while remaining wary of autonomy that substitutes clinician interaction—especially robot-led consent and fully robotic operations. Acceptance is stable across planned-procedure complexity; at ≥70 years, acceptance of tele-surgery is significantly lower (p=0.0017), while supportive vs autonomous preferences remain similar. Implementation in visceral surgery should prioritize surgeon-in-the-loop design, transparent patient communication (plain-language explanation of where and how AI is used), and robust privacy safeguards. As surgical AI scales, patient-centered preferences should inform pathway design, consent language, governance, and evaluation metrics—measuring not only accuracy and efficiency but also trust and acceptability along the continuum from augmentation to autonomy.

DE GRUYTER BRILL

**Evaluation of Molar pregnancy among reproductive age women in a tertiary teaching hospital**
ID: E-DCK-414

**Surgical Research**
**Clinical Studies**

Sheila Hares[1]

[1]SHEFAJO Specialized Private Hospital, Kabul, Afghanistan

**Background:**
A molar pregnancy is also known as hydatidiform mole which is a benign tumor that develops in the uterus. It begins when an egg is fertilized but normal viable pregnancy not occurs, rather than the placenta develops into an abnormal mass of cyst. There are two types of molar pregnancy: Complete and Partial molar pregnancy.

**Material and methods:**
A retrospective descriptive study was conducted in Shahr Ara teaching hospital, Kabul, Afghanistan over a period of three years (from 2022 to 2024). All reproductive age women (15-49 years old) who were diagnosed as molar pregnancy were included in the study.

**Results:**
In this study the incidence of molar pregnancy was 72 per7200 deliveries (1%). Among the patients 51 out of 72 (70.8%) were between (16 - 30 years) age group 18 out of 72 (25%) were between (31-40) and 3 out of 72(4.2%) were between (41-47years). Furthermore, 19 out of 72 (26.4%) cases were nulliparous 53 out of 72 (73.6%) were multiparous. About 94.5% patient covered by (D&C) and 5.5%treated by total abdominal hysterectomy (TAH). Among these we had just 7 recurrent patients (9.7%).

**Conclusion:**
Although molar pregnancy is rare, it poses significant risks to women and adversely impacts their quality of life. Early diagnosis and rigorous follow-up are critical to achieving high cure rates. Physicians must maintain a high index of suspicion to ensure prompt diagnosis, effective management, and close follow-up of the patients.
Health education plays a vital role in improving management and achieving favorable outcomes.

DE GRUYTER BRILL

**Exercise perfusion imaging with multispectral optoacoustics in PAD**
ID: E-DCK-514

**Surgical Research**
**Clinical Studies**

Briain Haney[1], Rabah Sankari[1], Ferdinand Knieling[2], Maximilian Waldner[3], Isabel Schöffel[2], Maximilian Dirauf[1], Ulrich Rother[1]

[1]Uniklinik Erlangen, Gefäßchirurgie, Erlangen, Deutschland, [2]Uniklinik Erlangen, Kinderklinik, Erlangen, Deutschland, [3]Uniklinik Erlangen, ;edizinische Klinik I, Erlangen, Deutschland

**Background:**
Non-invasive assessment of tissue oxygenation and perfusion during physical exertion offers novel opportunities for functional evaluation of muscle hemodynamics. This study aims to analyze the hemodynamic response of skeletal muscle during a threshold exercise test using multispectral optoacoustic tomography (MSOT). Differences between endurance athletes, strength athletes, non-athletes, and patients with peripheral arterial disease (PAD) are investigated, with a particular focus on intramuscular hemoglobin parameters.

**Material and methods:**
A total of 50 participants were enrolled in five groups of 10: endurance athletes, strength athletes, non-athletic young adults, patients with PAD, and age-matched physically active controls. All subjects performed a standardized threshold test on a treadmill-ergometer. Dynamic MSOT imaging of the anterior thigh musculature (m.vastus lateralis) was conducted to quantify oxyhemoglobin ($HbO_2$), deoxyhemoglobin (HbR) and MSOT derived Saturation (mSO2). Measurements were taken at rest, between the thresholds, and during the recovery phase. Results were analyzed on a group basis.

**Results:**
Endurance athletes demonstrated significantly higher overall mSO2 values and faster reoxygenation during the recovery phase between thresholds compared to strength athletes and non-athlete controls. PAD patients showed a markedly blunted $mSO_2$ response and delayed reoxygenation following exertion. In contrast, age-matched physically active individuals exhibited a hemodynamic profile comparable to that of the young endurance athletes.

**Conclusion:**
MSOT enables dynamic and detailed assessment of muscle perfusion during exercise. The findings reveal substantial differences in the hemodynamic response among the studied groups and highlight the potential of MSOT as a functional diagnostic tool for vascular diseases such as PAD, as well as for exercise monitoring in sports medicine. In the future, MSOT may serve as a monitoring tool for individualized training adjustments within structured walking programs for PAD patients, as well as training programmes in sports exercise.

DE GRUYTER BRILL

**Neoadjuvant Atezolizumab and Chemotherapy for Non-Squamous NSCLC: Results of a Phase II Trial**
ID: E-DCK-52

**Surgical Research**
**Clinical Studies**

Benedikt Niedermaier[1 2], Tilmann Bochtler[3], Florian Eichhorn[1 2], Pornpimol Charoentong[3 4 5 6], Raffaella Griffo[1 2], Laura V. Klotz[1 2], Michael Allgäuer[7], Albrecht Stenzinger[2 7], Maria Paula Roberti[3 4 5 6], Helge Bischoff[8], Marc A. Schneider[2 9], Petros Christopoulos[2 8], Uwe Haberkorn[2 10 11], Claus-Peter Heußel[2 12 13], Inka Zoernig[3 4 5 6], Felix J.F. Herth[2 14], Michael Thomas[2 8], Alexander Rölle[3], Svenja Schuehle[4 5 6], Julia Pollmann[3], Hauke Winter[1 2], Dirk Jaeger[3 4 5 6], Martin Eichhorn[1 2]

[1]Department of Thoracic Surgery, Thoraxklinik, Heidelberg University Hospital, Heidelberg, Deutschland, [2]Translational Lung Research Center (TLRC) Heidelberg, Member of the German Center for Lung Research (DZL), Heidelberg, Deutschland, [3]Department of Medical Oncology, Heidelberg University Hospital, Medical Faculty Heidelberg, Heidelberg University, Heidelberg, Deutschland, [4]Clinical Cooperation Unit Applied Tumor Immunity, National Center for Tumor Diseases, German Cancer Research Center, Heidelberg, Deutschland, [5]Center for Quantitative Analysis of molecular and Cellular Biosystems (Bioquant), Heidelberg University, Heidelberg, Deutschland, [6]National Center for Tumor Diseases (NCT) Heidelberg, Heidelberg, Deutschland, [7]Institute of Pathology, Heidelberg University Hospital, Heidelberg, Deutschland, [8]Department of Thoracic Oncology, Thoraxklinik, Heidelberg University Hospital, Heidelberg, Deutschland, [9]Translational Research Unit (STF), Thoraxklinik, Heidelberg University Hospital, Heidelberg, Deutschland, [10]Clinical Cooperation Unit Nuclear Medicine, German Cancer Research Center (DKFZ), Heidelberg, Deutschland, [11]Department of Nuclear Medicine, Heidelberg University Hospital, Heidelberg, Deutschland, [12]Department of Diagnostic and Interventional Radiology with Nuclear Medicine, Thoraxklinik, Heidelberg University Hospital, Heidelberg, Deutschland, [13]Department of Diagnostic and Interventional Radiology, Heidelberg University Hospital, Heidelberg, Deutschland, [14]Department of Pulmonology and Critical Care Medicine, Thoraxklinik, Heidelberg University Hospital, Heidelberg, Deutschland

**Background:**
Combining neoadjuvant immunotherapy targeting the PD-1/PD-L1 pathway with chemotherapy has significantly improved survival outcomes in resectable non-small cell lung cancer (NSCLC). However, no reliable biomarkers that predict response or therapeutic benefit have yet been established. The aim of this study is to evaluate the efficacy and safety of neoadjuvant atezolizumab plus carboplatin/nab-paclitaxel, as well as the potential of immune signature profiling to predict responses in patients with non-squamous NSCLC.

**Material and methods:**
The IReP trial is an investigator-initiated, open-label, single-arm, prospective, monocentric phase II trial (EudraCT No. 2020-000388-21). Patients with previously untreated, pathologically confirmed, non-squamous NSCLC in stage II, IIIA and select IIIB (T3N2 only) were treated with atezolizumab and carboplatin/ nab-paclitaxel for 3 cycles followed by curative intent surgery. Major pathologic response was defined as primary endpoint. To assess the local immunological micromilieu, biopsies were taken prior to the start of neoadjuvant therapy and from the surgical resection specimen.

**Results:**
20 patients with histologically confirmed pulmonary adenocarcinoma in TNM-stage IIA (n=1, 5%), stage IIB (n=7, 35%) and stage IIIA (n=12, 60%) were enrolled and treated according to the study protocol. 19 patients (95%) received the full neoadjuvant course of 3 cycles; one patient only received the first cycle due to serious adverse events including immune-related diarrhea and neutropenia. Overall, 151 treatment-related adverse events were recorded and 13 patients (65%) had treatment-related adverse events of grade 3 or higher. There were no grade 5 events. After completion of the neoadjuvant treatment, clinical assessment of tumor response according to RECIST criteria demonstrated partial response in 10 (50%) patients and stable disease in 10 (50%) patients; $^{18}$F-FDG PET/CT restaging showed a significant decrease in $SUV_{max}$ (15.1 vs. 4.2, $p < 0.001$) across the total cohort. Patient-reported outcomes according to the EORTC QLQ-30 questionnaire revealed a decline in global health status as well as physical and social functioning, but all patients were fit and with adequate function for surgery. Complete anatomical resection (R0) was performed in all patients. Major pathologic response was observed in 9 patients (45%), including 5 (25%) patients with complete pathological response. Pathologic response correlated moderately with the relative tumor size change in CT (r=0.522, p=0.018) and the relative change in $SUV_{max}$ (r=0.412, p=0.071). Pretherapeutic biopsies and surgical specimens are currently investigated for molecular and immunological biomarkers, immune cell subsets and the microbiome in relation to response.

**Conclusion:**
Neoadjuvant chemoimmunotherapy with atezolizumab is safe and achieves a promising MPR-rate. Clinical assessment of tumor response shows only moderate correlation with pathologic response. Predicting response remains a major clinical need. Further data are expected after completion of the follow-up period in late 2026.

DE GRUYTER BRILL

**Putative α7-selective ligands control trauma-induced cytokine release via α9* nicotinic receptors**
ID: E-DCK-117

**Surgical Research**
**Immune Therapy**

Mona Mobasher[1 2 3], Hina Andleeb[4], Arik Hone[5], Roger Papke[6], Andreas Hecker[1], J. Michael McIntosh[5], Veronika Grau[1], Karl Kirschner[2], Katrin Richter[1 3]

[1]Department of General and Thoracic Surgery, Laboratory of Experimental Surgery, Justus Liebig University Giessen, Giessen, Deutschland, [2]Department of Computer Science and Institute of Technology, Resource and Energy-Efficient Engineering (TREE), University of Applied Sciences Bonn-Rhein-Sieg, Sankt Augustin, Deutschland, [3]Department of Natural Sciences, Bonn-Rhein-Sieg University of Applied Sciences, Rheinbach, Deutschland, [4]School of Pharmacy, Concordia University Wisconsin, Mequon, Wisconsin, Vereinigte Staaten, [5]Department of Biology, University of Utah, Salt Lake City, Utah, Vereinigte Staaten, [6]Department of Pharmacology and Therapeutics University of Florida, Gainesville, Florida, Vereinigte Staaten

**Background:**
Nicotinic acetylcholine receptors (nAChRs) on immune cells are promising therapeutic targets for inflammatory diseases, such as trauma-induced systemic inflammation and inflammatory pain. We have recently implicated both α7 and α9* nAChRs (*denotes homomeric or heteromeric α9 nAChRs) as regulators of the cholinergic anti-inflammatory system (CAS) [1-3]. This study investigates the binding sites of putative α7-selective ligands on these receptors and their implications for trauma-mediated cytokine release.

**Material and methods:**
We examined the effects of classical (e.g., acetylcholine, nicotine), unconventional (phosphocholine), and putative α7-specific agonists (S24795, PNU-282987) on the ATP-induced release of pro-inflammatory cytokines interleukin (IL)-1β, IL-18, and IL-1α in lipopolysaccharide-primed human monocytic THP-1 cells and THP-1-derived macrophages. Electrophysiological two-electrode voltage-clamp measurements were conducted on *Xenopus laevis* oocytes expressing human α9α10 nAChRs. To gain atomic-level insights into ligand-protein interactions, molecular docking was performed on pentameric assemblies of the extracellular domains of the following homomeric human nAChR proteins using AutoDock 4.2.6: a) the crystal structure α7 (PDB ID: 7EKI), b) a modeled assembly of the homomeric α9 (PDB ID: 6HY7), and c) an α9-based homology model (Modeller) of homomeric α10.

**Results:**
In cytokine-release experiments, acetylcholine, nicotine, phosphocholine, S24795 and PNU-282987 efficiently inhibited ATP-mediated release of the pro-inflammatory cytokines IL-1β, IL-18, and IL-1α, while the ATP-independent cytokine release remained unimpaired. The inhibitory cholinergic effect was reversed by the specific antagonistic conopeptides [V11L;V16D]ArIB (α7 inhibitor) and RgIA4 (α9* inhibitor), indicating the involvement of nAChRs containing α7 and α9* subunits. Electrophysiological measurements further supported the interaction of the putative α7-selective agonists S24795 and PNU-282987 with human α9α10 nAChRs. Molecular docking simulations revealed that all tested ligands showed plausible binding modes in homomeric α7, α9, and α10 nAChR models at orthosteric sites around the C-loop region. Similar results were obtained for methyllycaconitine, an in previous CAS studies frequently used putative α7-selective antagonist.

**Conclusion:**
We identified binding sites and functional interactions of putative α7-selective ligands on α9* nAChRs. These results underscore the importance of considering α9* nAChRs when developing α7-selective ligands for immunomodulation. Moreover, our findings highlight that unconventional nAChRs containing α9* subunits effectively regulate the ATP-induced release of pro-inflammatory cytokines and are potential therapeutic targets for inflammatory diseases and pain.

**References:**
[1] Richter et al. 2022, Front. Pharmacol. 13:981276.
[2] Richter et al. 2023, Front. Immunol. 14:1140592.
[3] Richter et al. 2023, Neuropharmacol. 240:109717

DE GRUYTER BRILL

**No Difference in Postoperative Complications Among NSCLC Cohorts by Neoadjuvant Treatment Type**
ID: E-DCK-306

**Surgical Research**
**Immune Therapy**

Varun Gupta[1], Ka-Won Noh[2], Stefan Welter[3]

[1]University Hospital of Cologne, Clinic and Polyclinic for General, Visceral, Thoracic, and Transplantation Surgery, Cologne, Deutschland, [2]University Hospital of Cologne, Institute of Pathology, Cologne, Deutschland, [3]Lung Clinic Hemer, Hemer, Deutschland

**Background:**
To evaluate whether neoadjuvant therapy, including conventional platin-based chemotherapy and chemoimmunotherapy, influences the rate of postoperative complications in patients with non-small cell lung cancer (NSCLC) compared to those who did not receive any neoadjuvant treatment, using a propensity score-matched analysis.

**Material and methods:**
This is a retrospective analysis of prospectively recorded patient data from a tertiary care hospital in Germany, covering 401 cases from 2021-2022. After excluding cases due to lack of data and applying propensity score matching using the R package MatchIt, 72 matched pairs were included in the final analysis. The Clavien-Dindo classification was used for grading postoperative complications.

**Results:**
The demographic and clinical characteristics of the non-neoadjuvant (WNA), neoadjuvant chemotherapy only (NA-C), and neoadjuvant chemoimmunotherapy (NA-CI) cohorts after matching had non-significant q-values, demonstrating homogeneous matching (Table 1). Cumulatively, 48% of WNA cases showed postoperative complications compared to 50% and 42% of the NA-C and NA-CI cohorts, respectively ($q > 0.9$; Table 1). Postoperative complications were generally higher in the WNA group compared to the NA group, with varying grades: WNA had the highest rates in grade 3 (6%), while NA-CI had the highest rates in grade 2 (25%), and NA-C had the highest rates in grade 1 (33%). Additionally, there was no significant difference between the NA-C and NA-CI cohorts when analyzed in subgroups.

**Table 1. The demographic and clinical characteristics of the non-neoadjuvant (WNA), neoadjuvant chemotherapy only (NA-C), and neoadjuvant chemoimmunotherapy (NA-CI) cohorts before and after matching.**

| Characteristics | Before matching (*N* = 401) | | | | After matching (*N* = 72) | | | |
|---|---|---|---|---|---|---|---|---|
| | WNA (*N* = 340) | NA-C (*N* = 28) | NA-CI (*N* = 33) | *q* value | WNA (*N* = 48) | NA-C (*N* = 12) | NA-CI (*N* = 12) | *q* value |
| Age, *N* (%) | | | | 0.01 | | | | > 0.9 |
| ≤ 65 | 137 (40) | 20 (71) | 17 (52) | | 29 (60) | 6 (50) | 7 (58) | |
| > 65 | 203 (60) | 8 (29) | 16 (48) | | 19 (40) | 6 (50) | 5 (42) | |
| Sex, *N* (%) | | | | 0.2 | | | | > 0.9 |
| Male | 180 (53) | 18 (64) | 23 (70) | | 28 (58) | 6 (50) | 8 (67) | |
| Female | 160 (47) | 10 (36) | 10 (30) | | 20 (42) | 6 (50) | 4 (33) | |
| Body mass index, *N* (%) | | | | 0.7 | | | | > 0.9 |
| Non-obese (≤ 29.9) | 256 (75) | 23 (82) | 26 (79) | | 37 (77) | 9 (75) | 8 (67) | |
| Obese (≥ 30) | 84 (25) | 5 (18) | 7 (21) | | 11 (23) | 3 (25) | 4 (33) | |
| Cardiovascular risk factors, *N* (%) | | | | 0.7 | | | | > 0.9 |
| Absent | 119 (35) | 12 (43) | 10 (30) | | 20 (42) | 3 (25) | 3 (25) | |
| Present | 221 (65) | 16 (57) | 23 (70) | | 28 (58) | 9 (75) | 9 (75) | |
| Diabetes Mellitus Typ 2, *N* (%) | | | | 0.6 | | | | > 0.9 |
| No | 290 (85) | 26 (93) | 27 (82) | | 43 (90) | 11 (92) | 11 (92) | |
| Yes | 50 (15) | 2 (7) | 6 (18) | | 5 (10) | 1 (8) | 1 (8) | |
| COPD, *N* (%) | | | | 0.7 | | | | > 0.9 |
| No | 223 (66) | 19 (68) | 24 (73) | | 32 (67) | 8 (67) | 10 (83) | |
| Yes | 117 (34) | 9 (32) | 9 (27) | | 16 (33) | 4 (33) | 2 (17) | |
| History of second cancer, *N* (%) | | | | 0.2 | | | | > 0.9 |
| No | 300 (88) | 23 (82) | 32 (97) | | 45 (94) | 10 (83) | 11 (92) | |
| Yes | 40 (12) | 5 (18) | 1 (3) | | 3 (6) | 2 (17) | 1 (8) | |
| Localization, *N* (%) | | | | 0.2 | | | | > 0.9 |
| Upper lobe | 179 (53) | 14 (50) | 16 (48) | | 19 (40) | 4 (33) | 6 (50) | |
| Middle lobe | 14 (4) | 0 | 0 | | 2 (4) | 0 | 0 | |
| Lower lobe | 126 (37) | 8 (29) | 13 (39) | | 16 (33) | 3 (25) | 4 (33) | |
| Multiple lobes | 20 (6) | 6 (21) | 4 (12) | | 11 (23) | 5 (42) | 2 (17) | |
| Unspecified | 1 | 0 | 0 | | 0 | 0 | 0 | |
| Histology, *N* (%) | | | | 0.11 | | | | > 0.9 |
| Adenocarcinoma | 182 (54) | 7 (25) | 15 (45) | | 19 (40) | 3 (25) | 5 (42) | |
| Squamous cell carcinoma | 105 (31) | 15 (54) | 12 (36) | | 19 (40) | 7 (58) | 6 (50) | |
| Others | 53 (16) | 6 (21) | 6 (18) | | 10 (21) | 2 (17) | 1 (8) | |
| Clinical UICC stage before therapy, *N* (%) | | | | < 0.001 | | | | > 0.9 |
| IA- IB | 171 (50) | 0 | 3 (9) | | 4 (8) | 0 | 1 (8) | |
| IIA - IIB | 86 (25) | 3 (11) | 4 (12) | | 6 (13) | 1 (8.5) | 2 (17) | |
| IIIA - IIIC | 65 (19) | 23 (82) | 18 (55) | | 26 (54) | 10 (83) | 4 (33) | |
| IVA - IVB | 18 (6) | 2 (7) | 8 (24) | | 12 (25) | 1 (8.5) | 5 (42) | |
| Surgery type, *N* (%) | | | | < 0.001 | | | | > 0.9 |
| Atypical resection/Segmentectomy | 215 (63) | 1 (4) | 21 (64) | | 11 (23) | 1 (9) | 1 (9) | |
| Lobectomy | 110 (32) | 23 (82) | 7 (21) | | 23 (48) | 7 (58) | 7 (58) | |
| Pneumonectomy | 15 (5) | 4 (14) | 5 (15) | | 14 (29) | 4 (33) | 4 (33) | |
| Sleeve Resection, *N* (%) | | | | < 0.001 | | | | 0.6 |
| No | 318 (94) | 19 (68) | 25 (76) | | 47 (98) | 10 (83) | 10 (83) | |
| Yes | 22 (6) | 9 (32) | 8(24) | | 1 (2) | 2 (17) | 2 (17) | |
| Clavien Dindo, *N* (%) | | | | 0.6 | | | | > 0.9 |
| 0 | 204 (60) | 16 (57) | 18 (55) | | 25 (52) | 6 (50) | 7 (58) | |
| 1 | 50 (15) | 8 (29) | 4 (12) | | 9 (19) | 4 (33) | 2 (17) | |
| 2 | 69 (20) | 4 (14) | 10 (30) | | 11 (23) | 2 (17) | 3 (25) | |
| 3 | 11 (3.2) | 0 | 0 | | 3 (6) | 0 | 0 | |
| 4 | 4 (1.2) | 0 | 0 | | 0 | 0 | 0 | |
| 5 | 2 (0.6) | 0 | 1 (3) | | 0 | 0 | 0 | |
| Postoperative complication, *N* (%) | | | | 0.14 | | | | > 0.9 |
| None | 204 (60) | 16 (57) | 18 (55) | | 25 (52) | 6 (50) | 7 (58) | |
| Minor (Clavien Dindo 1-2) | 119 (35) | 12 (43) | 14 (42) | | 20 (42) | 6 (50) | 5 (42) | |
| Major (Clavien Dindo 3-5) | 17 (5) | 0 | 1 (3) | | 3 (6) | 0 | 0 | |

**Conclusion:**

This propensity score-matched analysis shows that neoadjuvant therapy, whether conventional platin-based chemotherapy or chemoimmunotherapy, does not significantly increase postoperative complications in NSCLC patients compared to those without neoadjuvant treatment. Similar rates and grades of complications across all groups suggest these therapies are not independent risk factors for postoperative complications. Neoadjuvant chemoimmunotherapy can be safely administered to NSCLC patients, supporting their use in clinical treatment planning.

DE GRUYTER BRILL

**S1PR4 deficiency affects acute/chronic colitis in a murine model differently**
ID: E-DCK-366

**Surgical Research**
**Immune Therapy**

Celine Hähnel[1], Christopher Pohl[2], Annabel Kleinwort[1], Theresa Kordaß[1], Silvia Ribback[3], Stephan Kersting[1], Tobias Schulze[1]

[1]Universitätsmedizin Greifswald, Klinik für Allgemeine Chirurgie, Viszeral-, Thorax- und Gefäßchirurgie, Greifswald, Deutschland, [2]Universitätsmedizin Rostock, Klinik und Poliklinik für Kinder- und Jugendmedizin, Rostock, Deutschland, [3]Universitätsmedizin Greifswald, Institut für Pathologie, Greifswald, Deutschland

**Background:**
Immunological, genetic and environmental factors contribute to the development of chronic inflammatory bowel diseases (IBD). The common pathophysiological pathway of IBD appears to be an impaired tolerance of the immune system to epitopes of commensal or pathogenic components of the intestinal flora or food components. Various immune cells have been assigned a role in these processes, and the involvement of different T cell entities and mononuclear cells has been extensively studied. Only recently, various B cell populations have been shown to be involved in the regulation of IBD development. We have recently shown that $S1PR_4$ mediated signalling impacts peritoneal B cell migration and the development of B cell follicules in a murine knock-out model. Interestingly, FTY720, a modulator of S1P signalling, attenuates colitis development in various murine models. In this study, we investigate the role of $S1PR_4$ in the development of acute and chronic intestinal inflammation.

**Material and methods:**
Acute and chronical colitis was chemically induced in $s1p_4^{-/-}$ and *wildtype (wt)* C57Black/6 mice oral DSS administration. Clinical disease activity was assessed daily. At day 12 (acute model) or day 52 (chronic model), mice were sacrificed and histopathological and immunohistochemical analysis as well as analysis of the local and systemic immune response were performed.

**Results:**
While $S1PR_4$ deficiency resulted both in a lower clinical disease severity and less pronounced histopathological changes in the model of acute colitis, $s1pr_4^{-/-}$ animals suffered from a more severe course of disease in the chronic colitis model. In the chronic disease model, plasmatic IgA levels as well as the number of plasma cells in the intestinal wall were increased. In contrast, the number of IL-10 secreting B cells in the intestinal wall was reduced in $s1pr_4^{-/-}$ animals. In the model of chronic colitis, there was a significant increase in local levels of pro-inflammatory cytokines.

**Conclusion:**
Inhibition of $S1PR_4$ mediated signalling attenuates acute inflammation in a murine model of acute colitis, while it accentuates histopathological changes in a model of chronic intestinal inflammation. The use of

S1PR4-specific agonists and antagonists in the appropriate clinical setting may thus represent a way of individually influencing the course of the disease.

DE GRUYTER BRILL

**Patient Empowerment Through Immersive Hospital Experiences Before Operations or Procedures**
ID: E-DCK-39

**Surgical Research**
**Innovative Surgical Techniques**

Zeynep Akbal[1], Moritz Queisner[1], Igor Sauer[1], Christopher Remde[1], Christoph Rüger[1], Axel Winter[1], Karl Eisenträger[1], Max Maurer[1]

[1]Charité Universitätsmedizin Berlin, Department of Surgery, Berlin, Deutschland

**Background:**
Hospital stays often cause emotional stress long before admission, with up to 80% of patients experiencing worries about treatment or hospitalization (Mavrogiorgou et al., 2023), often linked to feelings of lost control or discomfort (Sauer 2015, 3). Invasive measures such as surgery, anesthesia, or intensive care trigger particular fears, while many patient questions—about the hospital environment, procedures, anesthesia, or intensive care—remain insufficiently answered in routine consultations. Such stress is associated with poorer postoperative outcomes and higher mortality (Kassahun et al., 2022). To address this, the project "Patient empowerment through immersive hospital experience before operations or procedures" (PERISKOP) at Charité pilots two innovative 3D films (for anesthesia and ICU) in form of psychological preparation for patient.
The aim of PERISKOP is to use immersive 360° films, accessible via VR headsets in the clinic and through an online portal, to improve patient preparation at Charité – Universitätsmedizin Berlin. As an innovative approach to patient education, the applications are used for preparation of anesthesia and intensive care unit (ICU). Through these videos, patients can realistically experience key processes of their hospital journey and associated environments before admission. In the framework of patient empowerment, potentially anxiety-inducing situations are rendered tangible in advance to reduce fears of the unknown. The films enable interested patients to familiarize themselves with the clinical environment and treatment situations at Charité before their stay. As part of the pilot project, the films are offered to patients during their preparation at the hospital by medical and nursing staff. Within the films, doctors and nurses accompany patients from a first-person perspective through potentially anxiety-inducing situations. The 360° video format allows patients to experience their surroundings in real scale, in 3D, and with 360° sound—as if they were actually there. Spatial annotations within the videos display additional information, for example about medical devices. This provides a gentle introduction to the clinical environment and special treatment situations, such as procedures in anesthesia induction, the operating room, or the intensive care unit. Two pilot films are currently available: 1) immediate pre-operative preparation including anesthesia and 2) the stay in an intensive care unit (ICU), and the ICU study is ongoing.
The ongoing digital transformation substantially alters the dynamics of the patient-physician relationship. Emerging media formats and digital dissemination channels facilitate more direct communication with patients and harness the scalability of digital interventions. While VR has established efficacy in the therapeutic context of anxiety disorders, comparable strategies tailored to preemptively addressing anxiety and uncertainty during hospitalization, in the service of patient empowerment, remain underdeveloped. The described immersive patient communication platform supplements conventional educational practices and enables access to a diverse patient population. Its modular architecture supports progressive expansion, thereby offering sustained benefits and optimizing the patient experience throughout the surgical care continuum at Charité – Universitätsmedizin Berlin.

**Material and methods:**
The study is a supportive and complementary method to clinical procedures and does not offer medical treatment (see project description).
Experimental setup: Randomized, controlled pilot study with two groups (intervention group -in VR- vs. control group -without VR-).
Three measurement points:
T1: Before the medical consultation (control group) or the consultation supplemented with the intervention (intervention group).
T2: After the medical consultation (control group) or the consultation supplemented with the intervention (intervention group).
T3: On the last day of the hospital stay.

Primary endpoint:
Change in APAIS total score between the control and experimental groups over time
(difference in APAIS total score between T1 and T2 or T3).

Secondary endpoints:
• Change in patient anxiety measured using the APAIS questionnaire (anxiety subscale) from T1 to T2 or T3
• Change in patient information satisfaction measured using the APAIS questionnaire (information subscale) from T1 to T2 or T3
• Change in anxiety/nervousness level measured using the STAI questionnaire (state measurement) from T1 to T2 or T3
• Clinic-specific anxieties and associated depressive symptoms using the HADS (T3).
• Qualitative evaluation of the video experience based on random patient interviews.

Materials:
-Virtual Reality (VR) setup includes: VR Head Mounted Display (HMD), ICU film & Computer.
-Questionnaires

**Results:**
The ICU study is ongoing.

**Conclusion:**
As the data collection is still ongoing, we cannot draw conclusions, yet. We expect that the pre-operative anxiety levels and the anxiety surrounding the ICU experience decrease after VR intervention. Our hypotheses and expected results are listed below.
Main research question: Can immersive stereoscopic 360° video experiences in virtual reality, provided prior to surgical procedures, lead to a reduction of peri- and postoperative anxiety in patients?
Primary hypotheses:
-There are differences in the change of the Amsterdam Preoperative Anxiety and Information Scale (APAIS) total score from T1 to T2 between the control group and the intervention group.
-There are differences in the change of the APAIS total score from T1 to T3 between the control group and the intervention group.

DE GRUYTER BRILL

**A Sustainable 3D-Printed Curriculum for Microsurgical Training in Undergraduate Medical Education**
ID: E-DCK-465

**Surgical Research**
**Innovative Surgical Techniques**

Hena Sharifi[1], Jama Wahid[1], Hajera Khaleqi[1], Hans Christian Schmidt[1]

[1]Universitätsklinikum Hamburg-Eppendorf, Hamburg, Deutschland

**Background:**
Microsurgery represents one of the most technically challenging areas of modern surgery, demanding exceptional fine-motor control, spatial orientation, and hand-eye coordination. Conventional microsurgical training often involves use of animal tissues, which raises ethical concerns and can be difficult to access. Early introduction to realistic and cost-efficient training models provides students with an ethical and sustainable entry into microsurgery, in line with the “3R” principles (Replacement, Reduction, Refinement). Studies such as those conducted by Wayne et al. (2006) and McGaghie et al. (2011) have demonstrated that simulation tools improve procedural proficiency and psychological readiness of novice practitioners. In this vein, this approach helps reduce the use of animal materials.

**Material and methods:**
The course is designed for medical students in the clinical semesters who have already completed a basic suturing course. Each session spans four hours, including a break, and is conducted in small groups of three to four students to ensure individualized instruction. In collaboration with the Department of Ophtalmology at the University Medical Center Hamburg-Eppendorf (UKE), suitable facilities, microsurgical instruments, microscopes, and an operative simulator are provided.
Learners receive immediate and structured feedback from tutors trained in delivering cunstructive guidance. Progress is systematically monitored through tutor-student-interactions and self-assessment checklists, enabling participants to evaluate and improve their skills after each module. This structured approach supports the progressive development of microsurgical proficiency.
The course features three modules: (1) dexterity exercises to develop precise hand movements; (2) suturing on a self-developed "microsurgical playground," a model designed for practice; and (3) performing end-to-end anastomoses. Guided by trained tutors, students develop ergonomic working habits, safe handling of microsurgical instruments, and bimanual operation under the microscope. Complementing the hands-on learning, students also have access to digital learning materials, including, structured preparation scripts, and a clinical simulator.
Models are produced using polylactic (PLA) filament, a biodegradable plastic, and cast with silicone to simulate the elasticity and wall thickness of vessels. Printing is performed using a Bambu Lab X1 Carbon printer with a 0.4 mm nozzle. Design and adjustment are carried out in Autodesk Fusion 360, a computer-aided design (CAD) software. G-code preparation is done in Bambu Slicer, a 3D printing software that generates digital instructions for the printer. The course will run from December 2025 to Februeary 2026.

**Results:**

As the course has not started yet, no results are available. However, we have outlined specific performance metrics to measure anticipated skill gains. These include the time taken to complete standard microsurgery tasks, the accuracy of suture placement evaluated through standardized leak rate tests, and the use of validated rating scales to assess fine motor skills and hand-eye coordination. These quantifiable endpoints will form the basis for a detailed analysis of participant improvement once the course commences. The 3D-printed, silicone-based models should offer anatomical realism and avoid animal tissue use.

**Conclusion:**
The curriculum offers an innovative, ethical, and sustainable approach to undergraduate microsurgical training. The program integrated 3D-printed silicone models, tutor supervision, digital resources, and clinical simulation to facilitate effective early skill acquisition. While non-living models do not fully replicate the properties of living tissue, such as true elasticity and pulsatile flow, they offer a consistent and cost-effective platform for practice. The course enhances surgical education in pratical, didactic, and structural dimensions and has the potential for implementation in other medical faculties.

DE GRUYTER BRILL

**Early MASH in a human 3D liver spheroid model – from mechanism to intervention**
ID: E-DCK-251

**Surgical Research**
**Novel therapeutics, Biomarker, Targeted Therapy**

Niklas Bogovic[1], Philipp Kreiner[1], Hans Schlitt[1], Edward Geissler[1], Henrik Junger[1], Elke Eggenhofer[1]

[1]Uniklinik Regensburg, Klinik und Poliklinik für Allgemein- und Viszeralchirurgie, Regensburg, Deutschland

**Background:**
Early-stage metabolic dysfunction-associated steatohepatitis (MASH) is characterized by inflammatory, angiogenic, and fibrotic responses. Few preclinical models are available that reliably reflect the early features of MASH in human tissue. We established a multicellular 3D human liver spheroid system that recapitulates hallmarks of early MASH and enables pharmacological intervention testing.

**Material and methods:**
MASH spheroids composed of hepatocytes, hepatic stellate cells, sinusoidal endothelial cells, and Kupffer cells were cultured under control and MASH conditions. Inflammatory cytokines, endothelial adhesion molecules, angiogenic mediators, and fibrotic markers were quantified by ELISA on days 8 and 12 (n=4–10 independent pooled spheroid cultures per group, from ≥3 experiments). Collagen type III immunohistochemistry was performed to visualize fibrotic remodeling. Pharmacological modulation was assessed using the tyrosine kinase inhibitors Dasatinib and Nintedanib at three different concentrations. Statistical analysis was performed using non-parametric tests (Kruskal–Wallis with Dunn's correction).

**Results:**
MASH spheroids exhibited significant upregulation of IL-1β, TNF-α, ICAM-1, VEGF, and procollagen I, together with marked collagen III deposition. These findings reproduce key features of early MASH, including inflammatory activation, angiogenesis and fibrotic remodeling. Importantly, endothelial activation was consistent with early leakage phenomena, suggesting disruption of sinusoidal integrity. Pharmacological intervention demonstrated responsiveness: Dasatinib attenuated IL1β, TNF-α, ICAM-1 and procollagen I in a dose-dependent manner, while Nintedanib reduced VEGF and endothelial activation. Collagen III immunohistochemistry confirmed visible reduction of fibrotic remodeling under tyrosine kinase inhibition.

**Conclusion:**
This primary human 3D spheroid model captures the early phase of MASH, characterized by inflammatory, endothelial, and fibrotic signatures. The demonstration of pharmacological responsiveness highlights its translational potential as a platform for mechanistic studies and preclinical evaluation of targeted antifibrotic therapies.

DE GRUYTER BRILL

### Surgical Stress Meets Pulmonary Defense: The Role of Alveolar Macrophages in Neutrophil Activation

ID: E-DCK-352

**Surgical Research**
**Novel therapeutics, Biomarker, Targeted Therapy**

Carolin Rajewski[1], David Gallo[2], Leo Otterbein[2]

[1]Universitätsklinikum Hamburg-Eppendorf, Hamburg, Deutschland, [2]Harvard Medical School, Surgery, Boston, Vereinigte Staaten

**Background:**
Alveolar macrophages are considered the first line of defense against inhaled pathogens in the respiratory tract. As they recognize an invading bacterium, they become activated and rapidly secrete a number of cytokines and chemokines. Polymorphonuclear leukocytes (PMN) support macrophages in the appropriate immune response. One method to assess the role of the macrophage in immune response to bacterial challenge is to remove them from the environment. A promising approach that has been developed is specific genetic modifications that takes advantage of diptheria toxin (DT), which is toxic to cells. Mice do not have the receptor for DT and thus cells that can be engineered to express the diptheria toxin receptor (DTR) will be sensitive to the toxin, while neighboring cells will not. We used a mouse that is positive for DTR specifically on CD169+ (Siglec-1) cells, which include alveolar macrophages. The aim of this study was to 1.) characterize the effectiveness of this system and 2.) test whether depletion of airway macrophages altered the PMN recruitment in response to *Staphphyloccccus Aureus (SA)* bacteria and 3.) evaluate the impact on bacterial clearance in the lung.

**Material and methods:**
All mice received intratracheal DT. We next performed a bronchoalveolar lavage (BAL) on the mice to collect cells and fluid from the airway. Cell counts and cell differential were assessed in naïve animals and compared to those treated with DT for 24 and 72 hours. Seventy-two hours after DT, mice were treated with a sublethal dose of *SA* intratracheally. After waiting 4 hours, a BAL was performed. For further characterization of the macrophage phenotype in the lung, we completed immunofluorescent staining. Expression of the stress response protein Heme Oxygenase-1 was measured in the lung tissues by PCR.

**Results:**
Cells in the bronchoalveolar fluid are increased in both groups after administration of diphtheria toxin. However, the number of neutrophils exceeds the number of macrophages, which leads to the conclusion that macrophages were, in part depleted in the CD169 (Siglec1) DTR knock-in mice. After inoculation with *SA* there is a significant rise in neutrophils. Bacteria counts show that the CD169+ DTR mice clear *SA* from the airway better versus controls.

**Conclusion:**

The susceptibility to *SA* did not increase in the genetically modified mice as expected, instead we find that the DTR+DT mice were more effective in clearing the bacteria from the respiratory tract because the colony forming units were much less in spite of there being less macrophages present at the time of bacteria inoculuation.

DE GRUYTER BRILL

**Periportal Hotspot in MASH: Deep Learning–Enhanced Spatial Profiling**
ID: E-DCK-392

**Surgical Research**
**Novel therapeutics, Biomarker, Targeted Therapy**

Niklas Bogovic[1], Jens Werner[1], Philipp Kreiner[1], Hans Schlitt[1], Kirsten Utpatel[2], Henrik Junger[1]

[1]Uniklinik Regensburg, Klinik und Poliklinik für Allgemein- und Viszeralchirurgie, Regensburg, Deutschland, [2]Universität Regensburg, Institut für Pathologie, Regensburg, Deutschland

**Background:**
Metabolic (dysfunction)–associated steatohepatitis (MASH) represents an increasingly relevant comorbidity in hepatic surgery and transplantation. Its presence influences perioperative risk, yet the underlying spatial organization of inflammatory and fibrogenic signaling in human tissue remains insufficiently characterized. We examined whether pro-inflammatory cytokine transcripts accumulate within the periportal field, how these signals relate to periportal myofibroblast activation, and whether digital pathology approaches can provide robust, clinically applicable readouts.

**Material and methods:**
A total of 52 FFPE liver biopsies (controls: F0; MASH: ≥F1) were analyzed using RNAscope chromogenic in situ hybridization (CISH) for IL-1β, IL-6, TNF-α, and TGF-β. Periportal α-SMA immunofluorescence was quantified as the proportion of positive area. Regional transcript densities were extracted via QuPath and further refined using convolutional neural network–based Deep Learning to capture spatial heterogeneity and strengthen reproducibility. A composite inflammation index (z-IL-1β + z-IL-6 + z-TNF-α + z-TGF-β) was calculated. Routine laboratory values (ALT, platelets, INR) obtained at the time of biopsy were integrated into multivariable models.

**Results:**
MASH biopsies displayed higher periportal IL-1β, IL-6, and TNF-α signals compared with controls. Transcript density was consistently greatest in periportal regions, and periportal TGF-β aligned with α-SMA expression, indicating coupling of inflammatory and fibrogenic pathways. The composite CISH inflammation index rose progressively with fibrosis stage and differentiated MASH from controls. Its discriminative accuracy improved further when combined with laboratory parameters (AUC >0.80). Deep Learning–based analysis enhanced the detection of periportal hotspots and stabilized effect sizes across subgroups, identifying spatial interaction patterns not captured by manual assessment.

**Conclusion:**
This study demonstrates that periportal fields represent focal points of inflammatory and fibrogenic activity in MASH. By integrating RNAscope CISH, immunofluorescence, and Deep Learning–assisted image analysis, we established a reproducible, anatomically anchored signature that can be quantified objectively and combined with routine laboratory data. These findings support periportal-oriented biopsy evaluation, provide digital endpoints suited for surgical pathology workflows, and hold translational relevance for perioperative risk assessment in hepatic surgery and transplantation.

DE GRUYTER BRILL

**Identifying the appropriate organoid-based culture system for drug testing in PDAC**
ID: E-DCK-467

**Surgical Research**
**Novel therapeutics, Biomarker, Targeted Therapy**

Ayham Moustafa[1], Annika Brauer[2], Faik G. Uzunoglu[3], Thilo Hackert[3], Harriet Wikman[4], Christine Nitschke[3], Susanne Sebens[2]

[1]Laboratory of Radiobiology and Experimental Radiooncology, University Medical Center Hamburg-Eppendorf, Hamburg, Deutschland, [2]Institute for Experimental Cancer Research, University Hospital Schleswig-Holstein, Campus Kiel, Kiel, Deutschland, [3]Department of General, Visceral and Thoracic Surgery, University Medical Center Hamburg-Eppendorf, Hamburg, Deutschland, [4]Institute of Tumor Biology, University Medical Center Hamburg-Eppendorf, Hamburg, Deutschland

**Background:**
Pancreatic ductal adenocarcinoma (PDAC) is the 4th leading cause of cancer-related deaths in western countries, with an overall 5-year survival rate of less than 10%. Besides early metastasis, therapy resistance is a hallmark of PDAC, worsening the prognosis of patients. Resection of the tumor is limited to about 20 % of cases due to the advanced disease stage at the time of diagnosis, and chemotherapy (FOLFIRINOX, Gemicitabine/Paclitaxel) is used for adjuvant as well as palliative treatment. In PDAC, the tumor microenvironment (TME) can make up to 80% of the entire tumor mass. Predominant stromal cell populations are Cancer Associated Fibroblasts (CAF) and different immune cells, which can exhibit distinct (functional) phenotypes by which they may impact therapy responses. Recently, PDAC derived tumor cell organoids have been integrated into PDAC research, however, they still lack different stromal components and stroma heterogeneity. Many attempts have been made to uncover underlying molecular pathways and factors that govern resistance mechanisms. Here, global proteomic analysis has been performed to identify novel diagnostic markers and therapeutic targets of PDAC. Additionally, cell-selective proteomics have identified crucial proteins and cytokines involved in pre-metastatic niche formation associated with PDAC. However, despite these advances, the intricate molecular interplay between tumor cells and the heterogeneous TME remains to be elucidated.

**Material and methods:**
We aim to integrate our expertise in all participating institutions to achieve the following objectives:
1. To develop a stroma-enriched PDAC-derived organoid model to reliably identify patient-specific resistances
and sensitivities to defined chemotherapeutic drugs, which are applied as treatment for PDAC patients.
2. To systematically elaborate the maximum and minimum complexity in terms of stroma cell composition in
this organoid model to deliver a reliable prediction of drug responses in PDAC patients.
3. To perform quantitative proteomics analysis of untreated and treated organoid models with different complexities to uncover molecular pathways and factors involved in drug resistance, providing novel insights
into the complexity of the tumor stroma and therapy resistance of PDAC.
Tumor materials were obtained from PDAC patients. Out of these tissues, PDAC
organoids were established and co-cultured with CAF, peripheral blood mononuclear cells (PBMC), or both.
Drug testing was performed on the PDAC organoids under the different co-culture conditions using the patient-relevant drugs paclitaxel and gemcitabine.
Treatment response was assessed by M30 Cytodeath ELISA and automated microscopy.
Differential quantitative proteomics (bottom-up LC-MS/MS) was performed on all PDAC organoids under the various co-culture conditions.

**Results:**
Preliminary data of our pilot study on three selected organoid models showed:
The three selected models responded heterogeneously to the mono-cultures toward drugs.
The stroma enrichment differentially impacted the responses although not significantly. A total of 8,849 proteins were identified across all PDAC organoids.
Integrating the proteome of our PDAC organoids with the data of Collisson E A, et al. Heterogeneous proteome profiles were observed between mono-cultures and CAF-enriched cultures and depending on the different cultivation conditions.

**Conclusion:**
Outlook: We further aim to
• expand the organoids number and test additional cytostatic drugs.
• correlate experimental findings with clinical responses.
• test immunotherapy approaches.
• validate potential biomarkers and therapeutic targets.

DE GRUYTER BRILL

**Tractography of the basal ganglia predicts effects of DBS in Parkinson's Disease**
ID: E-DCK-512

**Surgical Research**
**Novel therapeutics, Biomarker, Targeted Therapy**

Ricardo Loução[1 2], Martin Kocher[3], Pablo Andrade[3], David Linden[4], Veerle Visser-Vandewalle[3]

[1]Klinik für Stereotaxie und Funktionelle Neurochirurgie, Uniklinik Köln, Köln, Deutschland, [2]Klinik für Allgemeine Neurochirurgie, Uniklinik Köln, Köln, Deutschland, [3]Klinik für Stereotaxie und Funktionelle Neurochirugie, Uniklinik Köln, Köln, Deutschland, [4]Department of Psychiatry, University Hospital Maastricht, Maastricht, Niederlande

**Background:**
Therapeutic approaches for Parkinson's disease (PD) patients include deep brain stimulation (DBS) of the subthalamic nucleus (STN). While the mechanisms underlying the therapeutic effects of STN-DBS are not completely understood, the observation that antidromic activation of the supplementary motor area (SMA) as a result of STN-DBS as inpired works on neurofeedback targeting the SMA. Using neurofeedback, a technique based on functional magnetic resonance imaging, patients can learn how to increase the brain activity of the SMA, which has been shown to lead to symptom reduction. In this work, we investigate pathways from the cortex, and within the basal ganglia, which are affected by STN-DBS and are linked to therapeutic effects, with the aim of expanding the potential candidates to be used as neurofeedback targets.

**Material and methods:**
We applied tractography to reconstruct the white matter pathways of the brain in each individual patient. Imaging data were acquired from PD patients scheduled for STN-DBS at our centre (N = 69). Clinical assessments like the UPDRS questionnaire were acquired before and after DBS surgery. We first developed an optimised fibre tracking pipeline for tracking in the basal ganglia. Afterwards, we investigated the connectivity of the volume of tissue activated (VTA) to the cortex in general and correlated the derived connectivity with the clinical outcomes. Then, we specifically targeted the connectivity within the cortico-basal ganglia-thalamocortical loop for tractography and calculated a degree of modulation for each pathway, by taking the proportion of affected fibres by the VTA, and these were correlated with clinical outcome.

**Results:**
VTA connectivity to the cortex revealed that VTA's primarily connect to the SMA. Correlation analysis revealed a positive correlation between motor clinical outcome and VTA connection to the SMA, pre-motor, and motor cortices. Positive correlations between the degree of modulation of individual pathways and clinical outcomes were identified, with the strongest ones being for the connection between STN and supplementary motor area (Spearman's rho = 0.28), STN and the globus pallidus internus (Spearman's rho = 0.29), and STN and the premotor areas (Spearman's rho = 0.29).

**Conclusion:**
This is, to the best of our knowledge, the largest study including patient-specific tractography of PD patients that underwent DBS to date. Our results show that connectivity within the basal ganglia also plays a role in the modulatory effects of DBS, potentially making nuclei like the pallidum a possible target for neurofeedback. Beyond that, the connectivity and pathway modulation profiles have the potential of building predictive clinical outcome models which could be used in a clinical context as a tool to guide therapy. The next steps will focus on validation using data from a partner centre, incorporation of non-motor symptoms into the analysis, and the development of symptom-specific predictive models.

Funding
Funds have been provided by the European Joint Programme Neurodegenerative Disease Research (JPND) 2020 call “Novel imaging and brain stimulation methods and technologies related to Neurodegenerative Diseases” for the Neuripides project ‘Neurofeedback for self-stImulation of the brain as therapy for ParkInson Disease’. The Neuripides project has received funding from the following funding organizations under the aegis of JPND: The Netherlands, The Netherlands Organization for Health Research and Development (ZonMw); Germany, Federal Ministry of Education and Research (BMBF); Czech Republic, Ministry of Education, Youth and Sports (MEYS); France, French National Research Agency (ANR); Canada, Canadian Institutes of Health Research (CIHR); Turkey, Scientific and Technological Research Council of Turkey (TUBITAK).

DE GRUYTER BRILL

**miR-155-5p and miR-3535 as pan-functional regulators of immune checkpoints and macrophages in PDAC**
ID: E-DCK-135

**Surgical Research**
**Oncology**

Theresa Kordass[1 2], Eric Nerenz[1], Vincencia Muliawan[1], Elena Knochenhauer[1], Aydar Khabipov[1], Stephan Kersting[1]

[1]Klinik und Poliklinik für Allgemeine Chirurgie, Viszeral-, Thorax- und Gefäßchirurgie, Universitätsmedizin Greifswald, Greifswald, Deutschland, [2]Pathologisches Institut UMM, Molekularpathologie, Mannheim, Deutschland

**Background:**
**Pancreatic ductal adenocarcinoma** (PDAC) remains one of the most lethal malignancies, with a five-year survival rate below 12% despite advances in surgery and systemic therapy. A hallmark of PDAC is its profoundly immunosuppressive tumor microenvironment (TME), which is largely shaped by tumor-associated macrophages (TAMs). These cells promote tumor progression, metastasis, and therapy resistance. Immune checkpoint molecules play a central role in this immune escape: adenosine-generating ectoenzymes such as CD39 (ENTPD1) and CD73 (NT5E) suppress anti-tumor immunity, while PD-L1 (CD274) interferes with T cell activation. Together, these mechanisms render PDAC highly resistant to current immunotherapies, including checkpoint inhibitors, and contribute to its poor prognosis. To overcome this barrier, novel therapeutic strategies and a deeper understanding of the molecular drivers behind this aggressive disease are urgently needed.

**Material and methods:**
Publicly available PDAC datasets (TCGA-PAAD, GTEx) were interrogated using GEPIA2 to assess expression and prognostic relevance of immune checkpoints and their correlation with miRNAs of interest. A panel of PDAC cell lines with varying baseline levels of CD73 (NT5E) and PD-L1 (CD274) was used for functional analyses. Cells were transfected with miRNA mimics (conventional lipid-based reagent), and surface expression of immune checkpoints was quantified by flow cytometry, while transcriptional changes were evaluated by qPCR. To assess effects on the immune compartment, monocyte-derived macrophages were generated from buffy coats of healthy donors and stimulated with miRNA mimics. In parallel, THP-1 monocytes were differentiated with PMA and polarized towards an M2-like phenotype using IL-4 and IL-10. Functional readouts included cytokine secretion (ELISA, Legendplex) and qPCR analysis of M1/M2 polarization markers. To advance translational potential, lipid nanoparticles (LNPs) were evaluated as delivery vehicles for miRNAs. Ongoing work includes testing these in 2D and 3D tumor and co-culture systems.

**Results:**
Analysis of PDAC datasets (TCGA-PAAD, GTEx) showed that NT5E (CD73) and ENTPD1 (CD39) are upregulated in tumors and that high NT5E correlates with poor survival. CD274 (PD-L1) expression was associated with adverse prognosis, while high MIR155HG expression correlated with favourable outcomes. Predicted miR-155-5p binding sites in NT5E and CD274, together with inverse correlations across cancers, support a regulatory interaction. Experimental validation in PDAC cell lines (e.g. PANC-01, MiaPaCa-2, PaTu-8988t/s) demonstrated that transfection with miR-155-5p mimics significantly reduced NT5E (CD73) surface expression, as measured by flow cytometry. In macrophage models, both **miR-155-5p and miR-3535** reprogrammed M2-like cells towards a pro-inflammatory phenotype, characterized by increased TNF-α and CXCL10 and decreased CCL22 mRNA levels.

**Conclusion:**
**miR-155-5p and miR-3535 act as pan-functional miRNAs** capable of downregulating immune checkpoints molecules on pancreatic cancer cells and reprogramming macrophages. Their dual activity highlights them as relevant regulators of the immunosuppressive tumor microenvironment. Future studies will further assess their effects on tumor cell growth and apoptosis. LNP-mediated delivery and testing in 3D co-culture models are being implemented to enhance translational relevance. A deeper understanding of the cell type–specific mechanisms underlying these regulatory effects may provide valuable insights into PDAC biology, support the development of miRNAs as prognostic or predictive biomarkers, and potentially open new avenues for therapeutic intervention.

DE GRUYTER BRILL

**Microbiome Diversity in Pancreatic Surgery: Preoperative Stenting and Postoperative Outcomes**
ID: E-DCK-172

**Surgical Research**
**Oncology**

Laura Oelschlägel[1], Johannes Klose[1], Markus Glaß[1], Stefan Moritz[2], Bogusz Trojanowicz[1], Artur Rebelo[1]

[1]Department of Visceral, Vascular and Endocrine Surgery, University Hospital Halle (Saale), Martin-Luther-University Halle-Wittenberg, Halle (Saale), Deutschland, [2]Department of Clinical Infectiology, University Hospital Halle (Saale), Martin-Luther-University Halle-Wittenberg, Halle, Deutschland

**Background:**
Carcinomas of the bile duct and pancreas remain highly lethal, with surgery being the only curative option. Despite reduced surgical mortality, postoperative complications prevail and adversely affect long-term survival. Over the last decade, a distinct microbiome has been identified in the pancreas and bile, previously considered sterile environments. However, microbial composition and clinical relevance remain unclear. We aimed to characterize the microbial communities across different body sites in patients undergoing pancreatic surgery and to investigate preoperative stent placement's effect on the microbiome as well as the influence of the most common species in this cohort on postoperative outcomes.

**Material and methods:**
224 samples (88 bile, 63 pancreatic fluid, 16 duodenal tissue, 35 cancer tissue, 22 healthy pancreatic tissue) were obtained from 58 patients during pancreatic surgery (11 bile duct cancer, 39 pancreatic cancer, 1 healthy pancreas, 7 chronic pancreatitis). The samples were analyzed using 16s rRNA gene sequencing. Clinical data were collected retrospectively. Alpha diversity was examined using the Shannon index, and differences were tested for significance using the Kruskal-Wallis-Test. For beta-diversity-analysis Non-metric Multidimensional Scaling (nMDS) and Permutational Multivariate Analysis of Variance (PERMANOVA) was used. Associations between the presence of the most common bacterial species in this study (≥20% of all samples, ≥1% mean relative abundance) and postoperative complications were assessed using binary logistic regression, adjusting for potential confounders (sex, age, body mass index, diabetes and smoking status).

**Results:**
Our analyses revealed distinct microbial profiles across body sites and diseases. Alpha-diversity differed when DNA- and RNA-based samples were analyzed separately. Beta-diversity analyses demonstrated a significant variation between duodenal, bile and pancreatic fluid samples (p=0.019), and between duodenal and pancreatic fluid samples from the same patient (p=0.017). In contrast, no significant differences were observed between tumor and adjacent healthy tissue or between bile and pancreatic fluid from the same patient. Among disease groups, significant beta-diversity differences were detected in tissue samples, but not pancreatic fluid, between pancreatic cancer and chronic pancreatitis (p=0.021), and between patients with and without preoperative biliary stents (p=0.001). Tissue samples from different carcinoma subtypes showed no significant variation. *Enterococcus faecalis* was associated with a reduced risk of severe postoperative complications (Clavien-Dindo ≥ III) (OR=0.89; 95% CI: 0.799-0.992; p=0.035). No significant associations were observed between the most prevalent species in this study and postoperative infectious complications. Risk-reducing (OR 0.17-0.89) and risk-increasing (OR 1.10-5.13) trends were identified, none reached statistical significance.

**Conclusion:**
This study indicates that microbial composition differs across body sites and pancreatic and biliary diseases, with biliary stent placement influencing beta-diversity. Alpha diversity analyses demonstrated the value of separate DNA-and RNA-based sample assessment. While the association of *Enterococcus faecalis* with a reduced risk for severe postoperative complications indicates a potential role of bacteria in surgical outcomes, no significant impact between other common species and postoperative complications was identified. Larger studies are warranted to validate these findings and identify potential targets that may support future therapeutic and diagnostic advances, enhancing overall prognosis.

DE GRUYTER BRILL

**AI-supported spatial multiplex immunofluorescence analysis of the Wilms tumor microenvironment**
ID: E-DCK-201

**Surgical Research**
**Oncology**

Nikolina Bradaric[1], Lukas Watzke[2], Francesca Palmisani[1], Katrin Rezkalla[1], Leo Kager[3], Michael Bergmann[4], Martin Metzelder[1], Helena Sorger[1]

[1]Medical University of Vienna, Department of Pediatric and Adolescent Surgery, Vienna, Österreich, [2]Medical University of Vienna, Department of Pathology, Vienna, Österreich, [3]St. Anna Children´s Hospital, Division of Hematology and Oncology, Vienna, Österreich, [4]Medical University of Vienna, Department of General Surgery, Division of Visceral Surgery, Vienna, Österreich

**Background:**
Wilms tumor is the most common pediatric renal malignancy, characterized by a triphasic histology composed of blastemal, epithelial, and stromal elements. The relative proportion and degree of maturation of these elements give a unique histological profile, contributing to variable tumor behavior and resulting in diverse clinical outcomes. Albeit widely recognized within the field of adult oncology, characterization of the tumor-stroma interplay was largely disregarded for pediatric solid tumors. Resolving the spatial organization, particularly of the stromal and immune populations within pediatric solid tumors, such as Wilms tumor, might provide insight into higher-level molecular and cellular processes that drive pathogenesis. However, such a complex analysis can only be accomplished using novel analysis tools, such as AI-supported image analysis, which enable advanced tissue annotations. Here, we aimed to spatially map the Wilms tumor TME using multiplex immunofluorescence and AI-supported tissue analysis to determine the distribution and density of activated fibroblastic and M2-like macrophage populations within the different tumor regions, at the tumor border and within healthy renal adjacent tissue.

**Material and methods:**
Formalin-fixed paraffin-embedded (FFPE) tissue sections from Wilms tumor patients (n = 46) operated on at the Department of Adolescent and Pediatric Surgery, Vienna General Hospital, in the period between 2002 and 2024, were collected and retrospectively analyzed. Patients were selected based on differing risk categories (high and intermediate), clinical outcome (relapse vs no relapse) and predominant histology types (blastemal, epithelial, mesenchymal, mixed and regressive tumor types) to create a cohort representing diverse tumor profiles. Tissue sections were processed for multiplex immunofluorescence (mIF) staining using well-established TME markers for myofibroblastic (a-SMA), general immune (CD45) and differentiated macrophage populations (CD163), as well as established and newly identified markers for different tumor regions. Tissue sections were imaged on the Vectra Polaris 1.0 (Akoya Biosciences, Inc.) and analyzed with quantitative image analysis software (HALO, Indica Labs). Area quantification was aided by the addition of the DenseNet AI classifier, which enabled detailed tumor annotations. Nonparametric ANOVA analysis was performed for statistical, using healthy renal adjacent tissue as the control group.

**Results:**

Based on DenseNet AI classifier training, tissue regions were successfully classified into healthy renal adjacent tissue, reactive stroma-rich or -poor regions, as well as blastemal, epithelial, mesenchymal, and regressive tumor areas with varying degrees of immune cell infiltration. Dense myofibroblastic populations (α-SMA$^+$ cells) were predominantly observed at tumor borders, forming a tight capsule particularly around blastemal tumor types, while their presence within the tumor areas was scarce and limited to mesenchymal (vimentin$^+$) tumor regions. Interestingly, M2-like macrophages (CD45$^+$ CD163$^+$), showed a similar pattern, predominantly residing in vicinity of α-SMA$^+$ fibroblasts and infiltrating into mesenchymal (vimentin+) tumor regions, with complete absence within blastemal regions. Importantly, patients who developed metastases or relapsed displayed significantly higher counts of M2-like macrophages in mesenchymal tumor areas (CD206$^+$, $P < 0.001$). Within the M2-like macrophage phenotype, a smaller population of vimentin$^+$ cells was observed, potentially representing a pre-trans-differentiation state.

**Conclusion:**
In this study, we spatially mapped activated fibroblastic and macrophage populations within distinct Wilms tumor and adjacent kidney regions, using multiplex immunofluorescence and AI-supported image analysis. The AI-supported analysis provides valuable opportunities to map the highly complex Wilms tumor TME by allowing advanced tissue annotations. We detected α-SMA$^+$ fibroblasts mainly at tumor borders and within vimentin$^+$ mesenchymal tumor regions. M2-like macrophages (CD45$^+$ CD163$^+$) were enriched in α-SMA$^+$ areas and infiltrated the vimentin$^+$ tumor mesenchymal regions, particularly within tumors from patients who relapsed or developed lung metastasis. Detection of a distinct vimentin$^+$ M2-like subset suggests presence of an alternatively activated phenotype, which should be further investigated. Based on the observed spatial organization, further studies will particularly focus on elucidating the immune-attracting, immuno-modulating and tumor-modulating effects of the detected fibroblastic populations. Overall, these findings highlight the prognostic potential of AI-supported TME characterization for pediatric renal tumors.

DE GRUYTER BRILL

**Differentiation and analysis of rectal tumor tissue using molecular spectroscopy**
ID: E-DCK-277

**Surgical Research**
**Oncology**

Luke Stratmann[1], Samuel Scheder[1], Anne Wedemann[1], Alexander Hirle[1], Marius Distler[1], Gerald Steiner[2], Christian Teske[1]

[1]Department of Visceral, Thoracic and Vascular Surgery, Medical Faculty and University Hospital Carl Gustav Carus, Technical University Dresden, Dresden, Deutschland, [2]Medical Faculty Carl Gustav Carus, Clinical Sensoring and Monitoring, Dresden University of Technology, Dresden, Deutschland

**Background:**
Currently the gold standard for intraoperative tumor margin assessment are fresh frozen cuts made by pathologists. They take approximately 20 minutes, are only looking at a small tissue slice at a time and can overlook small tumor cell clusters in other parts.
Colorectal cancer is currently the third most common tumor for men and women and the second most common cancer-associated death regardless of gender. An R0 resection with complete removal of the tumor brings a significant improvement in the overall survival.
IR spectroscopy offers a fast and label-free approach that has been used in many studies to analyse different tissues. Usually, with conventional methods, the tissue has to be preprocessed and the spectroscope used is bulky.
In this study we applied a small, mobile, fibre-based ATR spectroscope with high flexibility, enabling label free assessment of naive tissue within minutes.

**Material and methods:**
Freshly resected rectal tumor and normal tissue were measured with a fibre-based ATR module connected to a Bruker Alpha II Spectroscope. The tissue was first macroscopically classified by a pathologist, then measured and later histopathologically confirmed with cryocuts and HE staining. Feature extraction and supervised classification were performed with Matlab and correlated with histopathological diagnosis and patient data.

**Results:**
Preliminary results from an ongoing trial are promising. Mean spectra of 29 patients revealed distinct features and differences in the spectral regions 1080 $cm^{-1}$, 1188 $cm^{-1}$, 1250 $cm^{-1}$ and 1400 $cm^{-1}$. These areas are known to correspond to phosphodiester groups of nucleic acids (1080 $cm^{-1}$), deoxyribose (1188 $cm^{-1}$), amide III (1250cm-1) and $CH_3$ bonds of proteins and $COO^-$ groups of fatty acids (1400cm-1). A developed three-class classification model assigned tumor, normal, and metastatic tissue probability scores for class membership and accurately identified them.

**Conclusion:**
ATR IR spectroscopy is a fast and label-free way to accurately classify tissue as tumor, normal or metastatic tissue – even if the tissue is unknown.

This may aid in future surgical decision making, improve the workflow during the operation, reduce recurrence risk and therefore increase the overall survival of the patient.

DE GRUYTER BRILL

**Influence of obesity on tumour-defence mechanisms of mucosa-associated invariant T (MAIT) cells**
ID: E-DCK-281

**Surgical Research**
**Oncology**

Leiqian Xu[1], Katja Kotsch[1], Vanessa Pross[1], Najmeh Peyravi[1], Sarah Davoudzadeh[1], Ioannis Pozios[2], Pia Jäger[1 2 3]

[1]Charité Berlin Campus Benjamin Franklin, Allgemein- und Viszeralchirurgie - Chirurgisches Forschungslabor, Berlin, Deutschland, [2]Charité Berlin Campus Benjami Franklin, Klinik für Allgemein- und Viszeralchirurgie, Berlin, Deutschland, [3]Berlin Institute of Health at Charité – Universitätsmedizin Berlin, Berlin, Deutschland

**Background:**
Obesity is an independent risk factor for at least 13 cancer types and represents one of the most significant preventable causes of cancer. The increased cancer risk is partly due to immunological changes that impair tumour surveillance. T cells play a central role in recognising and eliminating precancerous and malignant cells. $CD8^+$ cytotoxic T cells are key effectors in direct tumour cell killing, while mucosa-associated invariant T (MAIT) cells also contribute to cytotoxic and regulatory functions.

MAIT cells can be activated via two mechanisms: T-cell receptor (TCR)-dependent recognition of microbial-derived riboflavin metabolites presented by MR1, and cytokine-mediated stimulation independent of TCR engagement. Upon activation, MAIT cells act at the crossroads of the adaptive and innate immune systems. They release pro-inflammatory cytokines such as IL-17 and IFN-γ, as well as cytotoxic effector molecules such as Granzyme B and Perforin. They also upregulate immune checkpoint receptors such as PD-1. MAIT cells express natural killer (NK) cell-like activating receptors, including CD161, NKp80, CD56 and the lectin-like receptor NKG2D, and are therefore capable of (mediating) cytolytic activity.

The prospective study *Project OncObesity* investigates the systemic and local effects of obesity and metabolic disorders on the functional capacity of MAIT cells in visceral adipose tissue (VAT) in the context of tumour defence.

**Material and methods:**
The immune cells of 45 patients, including 23 with clinical obesity (mean BMI: 47.93 vs. 27.09 in patients without obesity) and 14 with gastrointestinal cancer, are analysed. The cells are obtained from VAT and peripheral blood (peripheral blood mononuclear cells, PBMCs).

After processing the blood and tissue samples according to established protocols, PBMCs and VAT cells are isolated and analysed by flow cytometry. MAIT cells are identified by CD161 and Vα7.2 surface markers. For phenotypical analysis the expression of NKG2D, NKp80, and CD56 is analysed in unstimulated ex vivo MAIT cells. For functional analysis, flow cytometric analysis is performed after stimulation with PMA–Ionomycin.

DE GRUYTER BRILL

Statistical analysis is performed using parametric and non-parametric mean difference tests according to variable properties. For BMI-related correlations, correlation tests based on Pearson's product moment correlation coefficient are used. Adjusted analyses are performed using linear and generalised linear regression models.

**Results:**
We observe a significant increase in the frequency of MAIT cells in PBMCs when comparing individuals with a BMI of at least 30 to those with a BMI below 30 (mean 2.56% vs. 1.00%, $p = 0.032$). In multivariate linear regression analyses adjusting for sex, age, and cancer status, we identify a strong trend-level association between obesity and MAIT cell frequency in PBMCs ($R^2 = 0.30$, $p = 0.073$), whereas no such association is found in VAT. These findings suggest that obesity is associated with systemic alterations in MAIT cell frequency and tissue-specific distribution.

MAIT cells of patients with obesity display distinct phenotypic alterations, with correlations of surface marker expression for CD56 ($r = 0.25$), NKG2D ($r = 0.22$), and NKp80 ($r = 0.17$) on ex vivo cells.

The frequency of PD-1$^+$ MAIT cells is reduced in PBMCs in obese individuals and correlates negatively with BMI ($r = –0.2$, $p = 0.19$). However, no such changes are observable in VAT, suggesting that the functional state of MAIT cells is regulated in a tissue-specific manner in the context of metabolic alterations. This tissue-specific pattern is particularly evident within the subgroup of cancer patients ($n = 28$). Among this group, obese patients exhibit significantly lower frequencies of PD-1$^+$ MAIT cells in VAT compared to non-obese patients ($p = 0.038$). These results imply that the obesity-related increase in PD-1 observed in the VAT of non-cancer patients may not occur to the same extent in the presence of malignancy.

In functional analysis, Granzyme B expression in MAIT cells from VAT decreases significantly with increasing BMI ($\beta = –1.26$, $p = 0.027$), even after adjusting for age, sex, and disease type/comorbidity. This indicates reduced functional potential in individuals with obesity. These findings are consistent with those of the ex vivo phenotypic analysis, in which lower baseline Granzyme B expression is associated with higher BMI. The current data extend this observation by demonstrating that MAIT cells from individuals with obesity display impaired functional responses even upon stimulation. This suggests a persistent functional defect rather than merely a reduction in the activation threshold, thus reinforcing the concept of metabolically driven MAIT cell dysfunction in VAT.

**Conclusion:**
Our findings reveal an intricate relationship between metabolic dysregulation, obesity and the function of MAIT cells, both systemically and in adipose tissue. We observe obesity-associated alterations in the frequency and distribution of MAIT cells, with evidence of tissue-specific differences in phenotype and functional capacity. Notably, changes in the expression of activating and inhibitory receptors, including CD56, NKG2D, NKp80, and PD-1, suggest an altered immune regulatory state that is further influenced in the context of malignancy. Functional analyses following stimulation reveal reduced cytotoxic potential in MAIT cells from obese individuals, consistent with impaired effector responses. Taken together, these results support the hypothesis that obesity contributes to MAIT cell dysfunction, with potential implications for metabolic homeostasis and cancer immune surveillance.

DE GRUYTER BRILL

**Loss of caspase-3 leads to impaired cell proliferation**
ID: E-DCK-301

**Surgical Research**
**Oncology**

Michael Haase[1], Ivonne Kemnitz[1], Guido Fitze[1]

[1]Uniklinik Dresden, Klinik für Kinderchirurgie, Dresden, Deutschland

**Background:**
The activation of caspase-3 mediates cell death triggered by chemotherapy or radiation therapy. It is a common end-point of both extrinsic and intrinsic cell death pathways. However, it has been described that cell proliferation can be induced in the neighborhood of dead tumor cells or during sublethal activation of caspase-3. We wanted to further study this phenomenon in a cell culture model.

**Material and methods:**
We used a derivative of the chronic mylelogenous leukemia cell line KBM7 with and without CRISPR-Cas9 mediated knock-out (KO) of caspase-3. As a model for genotoxic injury, cells were UV-irradiated using a UV-crosslinker (Stratagene). Cells were counted with an CASY-Cell counter or with a Cellcyte microscope (ECHO). Proliferation-associated pathways were analyzed by Western Blotting.

**Results:**
Caspase-3 knock-out cells had a significantly reduced proliferation rate. Despite this MAPK1/3 (ERK p42/p44) activity was higher in the KO cells, demonstrated by a higher phosphorylation at threonine 202 (T202) and tyrosine 204 (Y204). Basal activation of p44 was very low but both subtype p42 and p44 were strongly activated in the KO cells. At 18 to 24 hrs after UV irradiation (UVR), both subtypes were maximally activated in KO cells. MAPK activation in control cells starts at 6 to 12 hrs after UVR and reaches maximal values at 18 to 24 hrs. Activation of MAPK14 (p38 MAPK) at T180 and Y182 is higher in KO cells. After UVR, MAPK14 is strongly activated in KO cells and moderately in Control cells. Activation of MAPK8/9/10 (JNK1/2/3) at T183 and Y185 is very low in non-irradiated cells independent of caspase-3 presence but is strongly activated after UVR after 15 min in KO cells and after 30 min in control cells both in the cytoplasm and in the nucleus. At 15 min after UVR, JNK is recruited to the cell membrane/organelle compartment in KO cells whereas this occurs after 30 min to 1 hr in control cells. In the non-irradiated state, P70S6 Kinase is more phosphorylated in KO cells, but after UVR, phosphorylation is later increased also in control cells.

**Conclusion:**
In this model of caspase-3 KO cells, there is a lower proliferation rate in the KO cells. The KO cells have a higher activation of cell-stress and proliferation-associated MAPK in the non-induced state and after UVR.

DE GRUYTER BRILL

### Multimodal approach predicts relapse upon cessation of immune checkpoint inhibitors in melanoma

ID: E-DCK-355

**Surgical Research**
**Oncology**

Ka-Won Noh[1], Yuri Tolkach[1], Doris Helbig[2], Varun Gupta[3], Reinhard Buettner[1], Oana-Diana Persa[4]

[1]University Hospital of Cologne, Institute of Pathology, Cologne, Deutschland, [2]University Hospital of Cologne, Cologne, Deutschland, [3]University Hospital of Cologne, Clinic and Polyclinic for General, Visceral, Thoracic, and Transplantation Surgery, Cologne, Deutschland, [4]Technical University of Munich, Munich, Deutschland

**Background:**
Treatment with immune checkpoint inhibitors (ICI) in advanced melanoma can result in durable responses, yet an algorithm to decide which patients can safely discontinue ICI is still lacking.

**Material and methods:**
We used a multimodal approach combining clinical data, AI-based analysis of H&E-stained whole-slide images of melanoma before ICI start, and gene expression signatures to identify biomarkers for relapse after discontinuing ICI in the absence of treatment progression.

**Results:**
Univariable Cox regression analysis identified best overall response, mRNA expression of six genes, tumor cell density (TCD), and the lymphocyte to plasma cell ratio (LYM/PC) as factors predictive of relapse upon cessation of ICI. Multivariable Cox regression analysis showed that both TGFBR1 expression and the integral digital pathology parameter-based prognostic system were independently associated with relapse after ICI discontinuation. Training a Multivariate Adaptive Regression Spline (MARS) model achieved the highest overall predictive accuracy of 84.6% for relapse after ICI discontinuation.

**Conclusion:**
The identified prognostic markers are fully explainable and easily implementable in routine practice and facilitate risk stratification upon cessation of ICI therapy.

DE GRUYTER BRILL

**Characterization of T Cell Exhaustion Signatures in Early-Onset Colorectal Cancer (EOCRC)**
ID: E-DCK-365

**Surgical Research**
**Oncology**

Josefine Schardey[1], Chao Li[1], Ulrich Wirth[1], Frank Braun[1], Jens Werner[1], Florian Kühn[1]

[1]Klinik für Allgemein-, Viszeral-, und Transplantationschirurgie, LMU Klinikum, München, Deutschland

**Background:**
T cell exhaustion (Tex)—a progressive dysfunction driven by persistent antigen stimulation—is a central mechanism of tumor immune evasion. However, the characteristics, clinical relevance, and diagnostic potential of Tex in early-onset colorectal cancer (EOCRC, <50 years) remain poorly defined.

**Material and methods:**
Peripheral blood mononuclear cells (PBMCs) were isolated and analyzed from 13 EOCRC patients, 24 propensity score–matched late-onset colorectal cancer (LOCRC) patients (matched for tumor localization, TNM stage, and grading), and 8 young healthy controls. Immunophenotyping was performed using multicolor flow cytometry. Differentially represented T cell subsets were identified and correlated with clinical parameters. Based on these data, we constructed a diagnostic model (univariate and LASSO regression). Findings were validated using publicly available bulk RNA-seq datasets from GEO (immune infiltration analysis) and single-cell RNA-seq data to characterize functional pathways and regulatory networks of Tex-associated genes.

**Results:**
EOCRC patients displayed distinct alterations in peripheral T cell populations compared to young healthy controls, including an overall increase in memory and exhausted T cells and changes in regulatory subsets, alongside a reduction of naïve and cytotoxic effector T cells. While the immune profiles of EOCRC and LOCRC were broadly comparable, EOCRC showed specific differences in $CD8^+$ T cell subsets, with a relative enrichment of exhausted ($CD39^+PD\text{-}1^+$) populations. Correlation analyses revealed that exhausted T cell signatures were associated with advanced tumor stage and elevated serum tumor markers, whereas cytotoxic T cells correlated negatively with disease burden.
Spearman correlation analysis revealed that exhausten markers on $CD8^+$ subsets ($CD39^+$, $TIGIT^+$, $TOX^+$) were positively associated with tumor stage and serum CEA, while cytotoxic $CD8^+$ T cells correlated negatively with tumor stage, CEA, and CA19-9. A diagnostic model derived from Tex and related subsets distinguished EOCRC patients from healthy controls with high accuracy.
Immune infiltration analysis of available GEO RNA-seq datasets confirmed a significantly reduced abundance of cytotoxic T cells and a downward trend of iTregs in EOCRC compared to young healthy controls, consistent with our flow cytometry findings. Single-cell RNA-seq identified 152 Tex-associated genes. Functional enrichment revealed their involvement in lymphocyte activation, antigen presentation, and immune receptor binding, as well as T cell differentiation, cytokine signaling, and inflammatory immune pathways.

**Conclusion:**
EOCRC patients display pronounced peripheral T cell exhaustion accompanied by impaired effector function. Alterations in circulating Tex subsets reflect the immune status of the tumor microenvironment and underscore their role in EOCRC-associated immunosuppression. Circulating exhausted T cells and related subsets may serve as biomarkers for EOCRC diagnosis and immune monitoring.

DE GRUYTER BRILL

**Pancreatic Tumor Dynamics in Real Time with the Chick Chorioallantoic Membrane Model**
ID: E-DCK-410

**Surgical Research**
**Oncology**

Andreas Ettner-Sitter[1 2], Benedikt Johannes Wagner[2], Silke Härteis[1], Christina Hackl[2]

[1]Department of Molecular and Cellular Anatomy, Universität Regensburg, Regensburg, Deutschland,
[2]Department of Surgery, University Hospital Regensburg, Regensburg, Deutschland

**Background:**
Pancreatic ductal adenocarcinoma (PDAC) remains one of the most aggressive and lethal malignancies, representing the fourth leading cause of cancer-related mortality worldwide. Despite advancements in diagnostic imaging and surgical techniques, the 5-year survival rate has stagnated at approximately 6%, largely due to late diagnosis, intrinsic chemoresistance, and the complex tumor microenvironment that characterizes PDAC. The desmoplastic stroma, composed of activated fibroblasts, extracellular matrix components, and aberrant vasculature, creates a hypovascular and hypoxic niche that profoundly influences tumor behavior and treatment response. Aberrant angiogenesis and vascular compression are hallmark features of PDAC progression, resulting in impaired perfusion, heterogeneous drug delivery, and increased interstitial pressure, all of which contribute to the poor therapeutic efficacy observed in clinical practice.
Given these challenges, there is a critical need for experimental models that accurately mimic the in vivo conditions of PDAC growth and angiogenesis while allowing dynamic, non-invasive monitoring of tumor behavior. Traditional murine xenograft models, although widely used, are costly, time-consuming, and raise ethical concerns due to the high number of animals required. In contrast, the chick chorioallantoic membrane (CAM) model represents a cost-effective, ethically favorable, and highly vascularized in vivo system that enables rapid engraftment and observation of human tumor tissues. The CAM, a naturally immunodeficient membrane of the chick embryo, provides an optimal microenvironment for human tissue xenotransplantation, supporting tumor vascularization, proliferation, and metastasis within a short experimental window.
In this study, we utilized the CAM model to investigate the dynamic progression of patient-derived PDAC tissue in real time. By integrating ultra-high frequency ultrasound imaging (71 MHz) and indocyanine green (ICG) angiography, we aimed to quantify tumor growth, vascularization, and perfusion longitudinally. Furthermore, histological and immunofluorescent analyses were employed to validate imaging findings and explore microvascular integrity, endothelial-tumor interactions, and the maintenance of tumor morphology. Through this combined approach, we sought to establish the CAM model as a viable platform for preclinical assessment of tumor dynamics and therapeutic responses, contributing to the development of personalized treatment strategies for PDAC patients.

**Material and methods:**
Human PDAC tissue samples were obtained from surgical resections following informed consent and ethical approval. Tumor fragments (~5 mm³) were grafted onto the CAM of fertilized chicken eggs at embryonic day 8 (EDD9), a developmental stage characterized by robust vascularization and sufficient membrane strength to support tissue implantation. After transplantation, embryos were incubated under controlled conditions (37.5°C, 60% humidity), and tumor development was monitored over seven days.

To assess tumor growth and vascular integration, **ultra-high frequency ultrasound imaging** at 71 MHz was performed daily, enabling high-resolution three-dimensional reconstruction of tumor morphology and volumetric quantification. This imaging modality allowed non-invasive tracking of tumor dimensions, surface area, and echogenic characteristics, reflecting internal structural changes and potential necrotic zones. Additionally, **ICG angiography** was employed to visualize perfusion and evaluate the functional connectivity between host and tumor vasculature. Indocyanine green was intravenously injected into the chorioallantoic vessels, and fluorescence imaging was used to assess dye penetration and perfusion kinetics within the grafted tissue.
For histopathological and immunohistochemical analyses, tumors were excised at EDD16, fixed in 4% paraformaldehyde, and embedded in paraffin. Sections were stained with **hematoxylin and eosin (HE)** for morphological evaluation, **CD31** for endothelial cell identification, and **cytokeratin 7** and **cytokeratin 19** to confirm the epithelial origin of human PDAC cells. Co-staining of Isolectin and human CD31 enabled visualization of perfused vessels and quantification of functional anastomoses between chick and human vasculature. Quantitative image analysis was performed using ImageJ software.
Therapeutic interventions were tested by intravenous administration of chemotherapeutic agents (e.g., FOLFIRINOX components) into the CAM vasculature. Tumor response was evaluated through serial imaging, volumetric assessment, and histological markers of apoptosis (cleaved caspase-3) and proliferation (Ki-67). Control groups received vehicle injections to establish baseline growth parameters.

**Results:**
PDAC tissue engraftment on the CAM demonstrated high viability and successful vascular integration in over 85% of cases. Within 24 hours post-implantation, host vessels began to migrate toward the graft, forming a dense peritumoral vascular network by day 3. **3D ultrasound imaging** provided reproducible quantification of tumor volume, enabling longitudinal assessment of growth dynamics across multiple scans. Mean tumor volume increased progressively over the first four days, followed by a plateau phase, suggesting adaptation to the microenvironment. Tumors displayed heterogeneous echogenicity, indicative of variable cellular density and early stromal formation.
**ICG angiography** revealed rapid perfusion of the tumor vasculature following intravenous dye injection, confirming the establishment of functional anastomoses between chick and human vessels. Histological evaluation corroborated imaging findings: HE sections revealed viable glandular structures surrounded by chick-derived capillaries, while CD31 staining identified integrated vascular networks bridging host and tumor domains. Co-localization of Isolectin and human CD31 confirmed functional perfusion through these hybrid vessels.
Immunohistochemistry for **CK7** and **CK19** confirmed the preservation of epithelial characteristics within the graft. Therapeutic application induced a reduction in tumor volume compared to controls, as quantified by ultrasound. Treated tumors exhibited decreased perfusion intensity and increased apoptotic markers, demonstrating the capacity of the CAM model to capture dynamic treatment responses in real time.
Notably, **ultra-high frequency ultrasound** enabled precise delineation of tumor boundaries and volumetric calculations. Serial imaging allowed continuous observation of tumor response, offering an advantage over endpoint-only analyses typical of murine models. The integration of imaging and histological validation established the CAM as a reliable system for quantitative assessment of tumor dynamics.

**Conclusion:**
This study demonstrates that the chick chorioallantoic membrane (CAM) model is a robust, cost-efficient, and ethically favorable platform for real-time analysis of pancreatic ductal adenocarcinoma (PDAC). Using high-resolution ultrasound and ICG angiography, we achieved non-invasive, longitudinal monitoring of tumor growth, vascularization, and therapy response. The CAM supports viable patient-derived PDAC tissue for up to seven days, allowing functional vascular integration and drug testing.
Compared to murine models, it offers faster experimentation, lower ethical impact, and compatibility with advanced imaging. Serving as a bridge between in vitro and in vivo systems, the CAM provides valuable insights into tumor biology and therapeutic response, contributing to the development of personalized treatment strategies for PDAC.

DE GRUYTER BRILL

**Integrative molecular risk signature in ductal cells responsible for malignant transformation.**
ID: E-DCK-500

**Surgical Research**
**Oncology**

Cao Zhong Jing Jin[1], Matthäus Felsenstein[1]

[1]Charité – Univsersitätsmedizin Berlin, Chirurgische Klinik, Berlin, Deutschland

**Background:**
Despite significant advances in the characterization of the molecular landscape of PDAC, clinically applicable biomarkers for early detection and risk-adapted intervention are still limited. A promising strategy for earlier diagnosis lies in the molecular stratification of pancreatic precursor lesions, particularly intraductal papillary mucinous neoplasms (IPMN). These lesions vary widely in their malignant potential, yet current clinical assessment relies primarily on radiological and histopathological criteria, which fail to capture biological heterogeneity. Notably, up to 11% of patients with IPMNs initially classified as low risk later develop PDAC. At the same time, single-cell molecular data from precursor lesions remain scarce compared to datasets from invasive carcinoma.

**Material and methods:**
This project aims to improve molecular risk assessment of precursor lesions immediately adjacent to invasive carcinoma by integrating single-nucleus RNA sequencing (snRNA-seq) and spatial transcriptomics (ST). This combined approach enables high-resolution analysis of gene expression profiles while preserving spatial context of relevant cell populations involved in tumor initiation and progression. The study includes 14 tissue samples from 13 patients who underwent pancreatic resection for PDAC with concomitant IPMN lesions at Charité – Campus Virchow-Klinikum, Berlin. Single nuclei were isolated from regions containing both carcinoma and directly adjacent precursor lesions, and sequencing was performed on the Illumina NovaSeq X system. Spatial transcriptomics profiling was conducted on matched tissue sections from eight cases using the NanoString GeoMx Digital Spatial Profiler (DSP).

**Results:**
In-depth bioinformatic analyses revealed a diverse cellular composition within the pancreatic tumor tissues, including epithelial, stromal and immune populations, as well as rare cell types not previously characterized. Ductal subpopulations across the spectrum of dysplasia were identified and transcriptional signatures associated with varying degrees of neoplastic transformation were identified. RNA velocity analysis is being applied to detect genes responsible for malignant progression in precursor populations. These transcriptional signatures will be integrated with ST data to map the localization of distinct ductal cell states within the tissue architecture.

**Conclusion:**

By correlating transcriptional profiles with spatial organization, this project aims to identify molecular markers predictive of tumor progression in precursor lesions. The resulting data have the potential to refine current risk stratification models on a molecular level and support the development of biomarkers for early detection and targeted surveillance. The innovation of this study lies in the combined application of snRNA-seq and ST to premalignant IPMN lesions directly adjacent to carcinoma, an area largely unexplored compared to invasive PDAC. This integrative design addresses key limitations of conventional histology- and imaging-based assessment. In the long term, these findings may facilitate improved patient selection for surgery, inform surveillance strategies, and contribute to precision medicine approaches in pancreatic oncology.

DE GRUYTER BRILL

**In vivo 3D tumor model to study human cholangiocarcinoma tissue**
ID: E-DCK-71

**Surgical Research**
**Oncology**

Agata Montagner[1], Andreas Ettner-Sitter[1], Benedikt Wagner[2], Thiha Aung[3], Silke Härteis[1], Christina Hackl[2]

[1]Lehrstuhl für molekulare und zelluläre Anatomie, Universität Regensburg, Regensburg, Deutschland, [2]Klinik und Poliklinik für Chirurgie, Universitätsklinikum Regensburg, Regensburg, Deutschland, [3]Fakultät Angewandte Gesundheitswissenschaften, Technische Hochschule Deggendorf, Deggendorf, Deutschland

**Background:**
Liver malignancies represent the third leading cause of cancer-related mortality worldwide (Sarcognato et al., 2021). After hepatocellular carcinoma, cholangiocarcinoma (CCA) is the second most common type of primary hepatic cancer, accounting for 10–15% of all hepatic carcinomas (Sarcognato et al., 2021; Elvevi et al., 2022). This malignancy carries a poor prognosis, largely due to delayed diagnosis and other factors (Izquierdo-Sanchez et al., 2022). While surgical resection is the only potentially curative treatment, many patients are not eligible (Cillo et al., 2019; Esmail et al., 2024). Moreover, treatment options for CCA patients are limited. These include the combination of gemcitabine and cisplatin, or capecitabine as adjuvant treatments (Primrose et al., 2019; Jeong et al., 2023). The combination of gemcitabine, cisplatin and PD-L1 inhibitor durvalumab is one of few treatment possibilities for neoadjuvant application and for inoperable patients (Oh et al., 2022; Yoo et al., 2023). In a palliative setting, FGFR inhibitors Pemigatinib and Futibatinib and the PD-1 inhibitor Pembrolizumab were also investigated (Vogel et al., 2024; Goyal et al.Kelley et al., 2023). Further molecular targets are currently tested in clinical trials. Given these new molecular targets on the one hand, and perioperative/adjuvant therapeutic limitations on the other hand, there is an urgent need to analyze these and new targets in novel models. Recently, three-dimensional tumor models have become increasingly used in cholangiocarcinoma research, with spheroids and organoids receiving particular attention. Most of these 3D models are in vitro systems, but one 3D in vivo model has recently begun to be applied in CCA research: the chorioallantoic membrane (CAM) model (Montagner et al., 2025). The CAM is a highly vascularized extraembryonic membrane of chicken eggs (Mapanao et al., 2021; Nowak-Sliwinska et al. 2014). This model is particularly versatile, allowing the cultivation of tumor cells and tissue, the testing of different drugs and the observation of angiogenesis (Ribatti et al., 2016). Additionally, this model significantly supports the reduction of animal experimentation in line with the 3R framework ("Reduce, Replace, Refine") (Tannenbaum et al., 2015). Our objective is to optimize the CAM model for cultivation of human CCA tissue.

**Material and methods:**
We cultivated human CCA tissue samples (n = 13) on the CAM, using both freshly resected specimens and cryopreserved samples; morphological changes were monitored by daily microscopic imaging. We studied the perfusion and tumor neoangiogenesis through two different methodologies: ultra-high-frequency ultrasound and laser speckle contrast imaging (LSCI). We performed ultrasound with a 70 MHz head to allow the visualization of also small capillaries on the CAM, with the aim of visualizing the perfusion of the tissue through vessels of the CAM. LSCI is a non-invasive optical technique for real-time blood flow imagining based on the creation of random interference pattern called “speckle” (Pion et al., 2023). We utilized LSCI measurement to evaluate the progressive perfusion of the tissue in a semi-quantitative way. We explanted the tissue grown on the CAM either after 7 days or sequentially every other day for time course analysis. Afterwards, we fixed the explanted tissue pellets in PFA embedded them in paraffin. We performed H&E histological staining for both primary tissue and tissue grown on the CAM, along with immunohistochemical staining for the proliferation marker Ki67.

**Results:**
We cultivated for the first time human CCA tissue on the CAM model. Both intrahepatic and extrahepatic CCA grew successfully on the CAM. Especially on day 7 of cultivation, we made the notable observation that CAM blood vessels invaded the engrafted tissue, albeit occasionally. LSCI measurements revealed increased vascularization of the CAM within the peritumoral region. Through ultra-high-frequency ultrasound measurements, it was possible to observe both the bigger blood vessels of the CAM and smaller capillaries supplying the tumor. Even after 7 days on the CAM, the tissue preserved typical histological features of CCA such as abnormal bile ducts and dense tumor stroma. We compared the proliferative activity before and after cultivation on the CAM through staining with Ki67 antibody. The tumor tissue on the CAM was still positive for Ki67, especially in areas of the tissue that were nearest to the CAM.

**Conclusion:**
This study provides the first evidence that the CAM model is suitable for the cultivation of human CCA tissue. Both intrahepatic and extrahepatic CCA samples were successfully cultivated for seven days, preserving key tumor characteristics and proliferative capacity. We achieved successful vascularization, and advanced imaging techniques confirmed increased tissue perfusion and neoangiogenesis. Importantly, the visualisation of the perfusion of the CCA tissue on the CAM provides proof of principle for future intravenous drug testing in this model. Moreover, the CAM system proved versatile, supporting both fresh surgical specimens and cryopreserved tissue, thereby offering experimental flexibility and broadening research applications. We achieved the objective of optimizing the CAM model for cultivation of human CCA tissue. The next aim will be to test chemotherapeutic drug intravenously on the CAM engrafted with human CCA tissue. Overall, the CAM model has strong potential to become a testing platform for researching various treatments on patient tissue, with the long-term aim of contributing to personalized therapy decision-making.

**Shear Stress–Induced IGF-1 From M2 Macrophages Accelerates Liver Growth: A Murine ALPPS Model.**
ID: E-DCK-188

**Surgical Research**
**Regeneration und Tissue Engineering**

Jan Heil[1 2], Simone Camargo[1], Birgit Roth Z'graggen[1], Jonah Neff[1], Martin Schläpfer[1 3], Beatrice Beck-Schimmer[1 3 4], Erik Schadde[1 5 6]

[1]Universität Zürich, Institut der Physiologie, Zürich, Schweiz, [2]Universitätsklinik Ulm, Klinik für Allgemein- und Viszeralchirurgie, Ulm, Deutschland, [3]Universitätsklinik Zürich, Institut für Anästhesiologie und perioperativer Medizin, Zürich, Schweiz, [4]University of Illinois at Chicago, Department of Anesthesiology, Chicago, Vereinigte Staaten, [5]Klinik Hirslanden, Chirurgisches Zentrum Zürich, Zürich, Schweiz, [6]Hirslanden St. Anna, VISCERA Lucerne, Luzern, Schweiz

**Background:**
In patients with an insufficient future liver remnant (FLR) at risk of posthepatectomy liver failure, regenerative liver surgery improves resectability by inducing FLR hypertrophy prior to resection. However, the mechanisms driving the accelerated regeneration observed in ALPPS (Associating Liver Partition and Portal Vein Ligation for Staged Hepatectomy) remain poorly understood. Previous work from our group identified hypoxia and shear stress as potential mediators of accelerated regeneration compared to the slower response following portal vein ligation (PVL). In this murine study, we investigated the role of liver-resident macrophages (Kupffer cells, KC) in accelerated liver growth, hypothesizing that KC contribute to shear stress–mediated regeneration in ALPPS.
Thirty C57BL/6 mice were randomized to ALPPS, PVL, or sham surgery with (-Cl) or without KC depletion using clodronate liposomes. Future liver remnant (FLR) volume of the right median lobe was assessed by contrast-enhanced micro-CT preoperatively and up to postoperative day (POD) 7. KC were quantified from FLR tissue, and their effect on hepatocyte proliferation was examined in vitro using immortalized KC and AML12 hepatocytes.

**Material and methods:**
Thirty C57BL/6 mice were randomized to ALPPS, PVL, or sham surgery with (-Cl) or without KC depletion using clodronate liposomes. Future liver remnant (FLR) volume of the right median lobe was assessed by contrast-enhanced micro-CT preoperatively and up to postoperative day (POD) 7. KC were quantified from FLR tissue, and their effect on hepatocyte proliferation was examined in vitro using immortalized KC and AML12 hepatocytes.

**Results:**
FLR growth was significantly greater in ALPPS (n=6) than in PVL (n=6), but was abolished in ALPPS-Cl (n=6) to levels comparable to PVL. In contrast, PVL and PVL-Cl (n=6) showed similar growth. In ALPPS at POD 3, the number of KC (CD68) and M2 (CD163) cells was significantly higher than in PVL. In vitro, shear stress–stimulated M2 macrophages secreted insulin-like growth factor-1 (IGF-1), which enhanced hepatocyte proliferation in response to hepatocyte growth factor (HGF). In vivo, differences in IGF-1 receptor signaling distinguished ALPPS from PVL.

**Conclusion:**
M2 macrophages appear to play a pivotal role in rapid liver regeneration following ALPPS by amplifying HGF-driven hepatocyte proliferation through shear stress–mediated IGF-1 release. Further studies are warranted to confirm the dependence of accelerated liver regeneration on IGF-1 signaling.

DE GRUYTER BRILL

**Near to nature? Maintained marker expression in aortic valve culture in a microphysiological system**
ID: E-DCK-199

**Surgical Research**
**Regeneration und Tissue Engineering**

Claudia Dittfeld[1], Anna Scheer[1], Maximilian Winkelkotte[1], Florian Schmieder[2], Stephan Behrens[2], Juergen Philipp[2], Antje Schauer[3], Anett Jannasch[1], Frank Sonntag[2], Sems-Malte Tugtekin[1]

[1]Faculty of Medicine and University Hospital Carl Gustav Carus, Technische Universität Dresden, Heart Centre Dresden, Germany, Department of Cardiac Surgery, Dresden, Deutschland, [2]Fraunhofer Institute of Materials and Beam Technology IWS, Dresden, Deutschland, [3]Faculty of Medicine Carl Gustav Carus, Heart Center Dresden, Technische Universität Dresden, Laboratory of Molecular and Experimental Cardiology, Department of Internal Medicine and Cardiology, Dresden, Deutschland

**Background:**
Degenerative alterations of the valvular extracellular matrix (ECM) are hallmarks of tissue pathophysiology of calcific aortic valve disease (CAVD) resulting in aortic valve stenosis. This is associated with dystrophic calcification. Nevertheless, especially active, cell-regulated, multifactorial processes are research foci and especially neoosteogenesis after activation and differentiation of valvular interstitial cells is responsible for disease progression. Impact of pathological ECM-remodeling with fibrotic thickening of the AV tissue on altered supply of oxygen and nutrients is hypothesized to promote tissue pathology. Monolayer 2D VIC culture and available small animal models are limited in pathogenesis reflection and organ-on-a-chip based tissue culture concepts are in development and already used to overcome limitations and complement results achieved. A microphysiological System (MPS) implementing a tissue incubation chamber (TIC) to realize dynamic, pulsatile culture of porcine AV tissues was used to investigate aortic valve subsegments. Dysregulated marker expression allows to monitor pathological processes in tissue culture AV samples and the investigation of substance-based treatment strategies can be performed in adapted MPS culture in future. Aim of the presented dataset is the evaluation of fibrosis or neoosteogenesis induction dependent on simulation of biomechanical properties in pulsatile dynamic aortic valve tissue culture.

**Material and methods:**
Porcine AV segments (pAVs) were cultured in a low flow- and high flow-setup in a pulsatile-dynamic TIC-MPS for 14 days (static and dead cell control implemented) using different pump systems. Tissue viability was evaluated via LDH- or Resazurin-assay, respectively. The mRNA expression profile of fibrosis, hypoxia or osteogenesis markers (COL1A1, ACTA2, FN1, TGF-ß1, HIF1α, RUNX2 and SOX9) was analyzed. Biomechanical MPS simulation was monitored by particle image velocimetry (PIV) to measure flow parameters with resulting calculation of shear forces and a rat pressure-volume catheter to detect pressure amplitudes.

**Results:**
In the MPS expression of HIF1α, COL1A1, ACTA2, TGF-ß1, and RUNX2 was significantly or with a tendency lower in pAV tissue cultures compared to the static culture and comparable to the native aortic valve tissues. In contrast, differences comparing low flow and high flow setup were marginal. VIC viability was comparable in tissue subsegment culture independent from static or dynamic setup. Adapting the pump chip to high flow setup resulted in a sixfold increase of the average flow rate to 77.4 µl/s in a TIC with a diameter of 6 mm. Therefore, shear force peak is elevated from 0.04 dyn/cm$^2$ in the low flow condition to 0.26 dyn/cm$^2$. High flow setup allowed the adaption of pressure amplitude from 68.5 ± 17.7 mmHg in the low flow setup to 53.1 ± 11.3 mmHg in the high flow setup due to the implementation of an attenuator element in the MPS.

**Conclusion:**
The pulsatile, dynamic culture of AV tissue segments in the MPS better preserves native tissue mRNA-expression values than statically cultured counterparts independent from still limited adaption of flow and pressure parameters in the TIC-MPS. Nevertheless and ongoing, approximation of physiological conditions in the MPS is envisioned. Results have to be substantiated by protein expression analyses and functional investigation of AV tissue calcification induction.

DE GRUYTER BRILL

**The rosetta stone of successful ageing: does oral health have a role?**
ID: E-DCK-261

**Surgical Research**
**Regeneration und Tissue Engineering**

Maximilian Poser[1]

[1]Universitätsklinikum Leipzig, Mund-, Kiefer- und Gesichtschirurgie, Leipzig, Deutschland

**Background:**
The attainment of very old age and accumulation of life experiences has always been fascinating to humankind. Throughout history humans have revered longevity. Elevated status of elders and ancestors in the late neolithic period or the pronounced role in culture (Xiao ), religion (James and Version ) or society (P C ) illustrate that point.
Research continues to examine the question: How do people achieve exceptional longevity and age successfully? Although many questions remain to be answered, metabolic dysregulation (Paolisso et al. ), inflammation (Arai et al. ; Franceschi et al. ), altered immune function (Salvioli et al. ; Wikby et al. ), endocrine changes (Paolisso et al. ; Suzuki et al. ) and increased oxidative stress (Belenguer-Varea et al. ) all seem to play a significant role in ageing. Social relationships (Holt-Lunstad et al. ), genetic background (Terry et al. ), posttranslational modifications (Ben-Avraham et al. ) and the interaction between our genes and the environment(Ebert et al. ; Christensen et al. ; Herskind et al. ) also appear to have a large impact. Previous research has partially uncovered some influential factors, however the entire complexity of attaining extreme longevity remains elusive.
Furthermore, the factors that influence extreme longevity have received different amounts of attention, with some influential factors having been omitted. One of the neglected areas could be the importance of oral health in achieving very old age. The following narrative review will illustrate the current research in the oldest old and discuss a missing piece of the puzzle, that might help to gain a more comprehensive and deeper understanding.

**Material and methods:**
This narrative review aims to summarize the current state of the literature hoping to unveil new perspectives and avenues for future research.

**Results:**
The maintenance of homeostasis is critical for extreme longevity, as aging involves complex interactions between genetic, lifestyle, and social factors. These interactions impact aging processes either directly or through mechanisms outlined in the hallmarks of aging. According to López-Otín et al., primary hallmarks include genomic instability, telomere attrition, epigenetic changes, and loss of proteostasis, which result from bodily insults. Antagonistic hallmarks—such as deregulated nutrient sensing, mitochondrial dysfunction, and cellular senescence—serve as compensatory mechanisms but eventually harm cellular function. Integrative hallmarks, like altered intercellular communication and stem cell exhaustion, arise from accumulated damage and insufficient regenerative responses. Recent studies have introduced inflammation and altered cellular mechanics as new hallmarks.

Oral health plays a significant role in aging by influencing nutritional intake and overall health. Inflammatory processes linked to dental pathologies can impair chewing function, affecting protein intake and potentially contributing to frailty and mortality. Periodontal disease, common in the elderly, is characterized by inflammation, and its management may reflect the body's broader ability to handle inflammation. An improved capacity to manage low-grade inflammation could be key to achieving longevity. Overall, maintaining oral health, along with healthy lifestyle behaviors like exercise and diet, may act as pillars supporting resilience and successful aging.

**Conclusion:**
This review illustrates that oral health plays a significant role in reaching exceptional longevity, especially considering its influence on nutrient uptake (Wolfe et al. ; Coelho-Júnior et al. ; Motokawa et al. ). Ageing research has greatly advanced following the landmark paper describing the hallmarks of ageing by establishing a common language and concept of the ageing process (Guevara-Aguirre et al. ; Gems and Magalhães ) Aspects of the ageing process have been examined in centenarians, most notably the impact of chronic inflammation (Arai et al. ). Inflammation plays a key role in the pathogenesis of periodontal disease and its' relation to other systemic comorbidities, (Zanni et al. ; Gondo et al. ) however the link between oral health and successful ageing remains underexplored. More research is needed to get a deeper understanding of the relationship between oral health and other processes known to play an important role in ageing. Translating and synthesizing this knowledge will be a challenge, but must include dental research in order to see the full picture. Similar to the Greek on the rosetta stone, which acted as the key in deciphering the hieroglyphs, oral health could be a translational piece to understand the process of ageing.

DE GRUYTER BRILL

**Adhesive Silicon-based Cell Culture System for Differentiation of Human Adipose Tissue-derived MSC**
ID: E-DCK-347

**Surgical Research**
**Regeneration und Tissue Engineering**

Peggy Stock[1], Janis Hildebrand[2], Bruno Christ[1], Tim Ricken[3], Gunter Böttcher[2], Hans-Michael Tautenhahn[1]

[1]University of Leipzig Medical Center, Visceral, Transplant, Thoracic und Vascular Surgery, Leipzig, Deutschland, [2]KET Kunststoff- und Elasttechnik GmbH, Radeberg, Deutschland, [3]University of Stuttgart, Institute of Structural Mechanics and Dynamics in Aerospace Engineering, Stuttgart, Deutschland

**Background:**
Conventional two-dimensional as well as recently established three-dimensional cell culture systems exhibit a high modulus of elasticity, yet, without representing the stiffness of human organs. Previously, we established a protocol for the culture of primary rat hepatocytes on a silicone of low stiffness, which sustained their physiological functions. This tool may readily serve as validation system for pharmacological and toxicological studies of agents and compounds. In terms of the development of human-based tests to evaluate the potential risk of novel drugs, the method was adjusted for use of human adipose tissue-derived mesenchymal stromal cells (hAT-MSC).
Aim of the study was to optimize culture conditions for hAT-MSC on fibronectin- coated silicone layers with various elastic moduli (50 – 500 kPA) mimicking the stiffness of human organs including different surface modifications to increase hydrophilic properties.

**Material and methods:**
The metabolic activity (MTT assay) and growth parameters were analysed and compared to the conventional cell culture on polystyrene (E-modulus 103 MPa). hAT-MSC were cultured for 10 days on five different fibronectin-coated medically approved silicone layers. The impact of surface modifications of the silicone using sol-gel-technique, fluorination and plasma treatment on the cells was determined by the development of cell counts, viability and metabolic activity.

**Results:**
hAT-MSC attached to all 5 different silicone layers and formed cell layers after 10 days. Neither the silicones with surface modifications nor the untreated silicone displayed significant differences in cytotoxicity: the percentage of dead cells in cultures were 7% ± 2.3% vs. 7.2% ± 1%. We identified one culture displaying significantly higher cell counts as compared to the standard culture: based on identical initial cell counts, the growth rate was about 2.5-fold higher on plasma-treated silicone with an E-modulus of 50 kPa. Cells cultured on plasma-treated silicones with E-moduli of 50 kPa or 500 kPa showed also significantly higher amounts of metabolic activity as compared to the classical culture: 2.76-fold (50 kPA) and 2.83-fold (500 kPa).

**Conclusion:**

With these results, two medically approved silicones treated with plasma were identified for improving culture of hAT-MSC. Thus, the silicone layers represent an important tool for the development of cell cultures featuring organ-like stiffness and improving physiological functions.

European Regional Development Fund and the Free State of Saxony (SAB) 100282466.

DE GRUYTER BRILL

**Macrophage Enrichment Drives Inflammation and Lung Hypoplasia in Congenital Diaphragmatic Hernia**
ID: E-DCK-44

**Surgical Research**
**Regeneration und Tissue Engineering**

Richard Wagner[1], Jan Riedel[1], Katinka Sturm[1], Jan-Hendrik Gosemann[1], Martin Lacher[1]

[1]Universtiätsklinikum Leipzig, Klinik und Poliklinik für Kinderchirurgie, Leipzig, Deutschland

**Background:**
Congenital diaphragmatic hernia (CDH) is a life-threatening congenital anomaly characterized by pulmonary hypoplasia and pulmonary hypertension, which remain the major determinants of survival. The etiology of lung hypoplasia in CDH is multifactorial and incompletely understood. While impaired branching morphogenesis and vascular remodeling are well established, recent work has highlighted a role for inflammatory signaling pathways in abnormal lung development. In particular, NF-κB–dependent transcriptional activity was shown to be hyperactivated in epithelial cells derived from human CDH neonates and in fetal lung sections from CDH patients. However, the cellular mediators of this inflammatory milieu have not been fully defined. Based on preliminary findings in animal models, we hypothesized that pulmonary macrophages represent central regulators of the inflammatory microenvironment in hypoplastic CDH lungs.

**Material and methods:**
To address this, we combined human and experimental data across multiple complementary platforms. Proteomic profiling was performed on human fetal CDH lungs (n=6, 30–36 weeks' gestation) and matched controls (n=6) using label-free quantitative mass spectrometry. Pathway enrichment analysis and gene ontology clustering identified biological processes linked to immune activation. In parallel, tracheal aspirates from CDH neonates collected immediately after birth were analyzed. Immunohistochemistry and quantitative image analysis were used to determine macrophage density in formalin-fixed lung sections from human CDH fetuses compared with controls. Finally, the nitrofen rat model of CDH was used to define temporal dynamics of macrophage infiltration and test whether anti-inflammatory treatment can improve lung development in CDH.

**Results:**
Proteomic profiling of human fetal CDH lungs revealed significant enrichment of innate immune pathways, with overrepresentation of proteins involved in macrophage activation and NF-κB signaling. Among the most discriminant proteins separating CDH from controls were AGER, RAC1, and PLS3, all previously implicated in immune and cytoskeletal regulation. Gene ontology analysis identified clusters related to activation of innate immune responses, defense against pathogens, and viral response, highlighting a proinflammatory state. Tracheal aspirates from CDH neonates further supported this finding, showing elevated levels of macrophage chemoattractants, compared to non-CDH controls. These data indicate that inflammatory cell recruitment is active in CDH not only prenatally but also immediately after birth. Histological analysis of human fetal CDH lungs confirmed an increased density of macrophages compared with gestational age-matched controls. In the nitrofen rat model, macrophage infiltration was already evident at E15, preceding overt structural lung hypoplasia. At E21, macrophage numbers were further increased, affecting both left (ipsilateral) and right (contralateral) lungs. Moreover, blockade of pro-inflammatory signaling restores normal epithelial differentiation phenotypes in human CDH BSCs in vitro and improves lung development in the nitrofen CDH model in vivo.

**Conclusion:**

This work establishes pro-inflammatory signaling and macrophage enrichment as a hallmark of abnormal lung development in CDH across species, developmental stages, and biological compartments. The integration of human proteomic data, neonatal tracheal aspirates, fetal histology, and the nitrofen model provides compelling evidence that macrophage-driven inflammation is an important factor in lung hypoplasia. Macrophages were enriched bilaterally in nitrofen-exposed fetuses, underscoring the systemic nature of the defect. The close correlation with epithelial NF-κB activation in both human and rat CDH lungs provides mechanistic insights into how innate immunity disrupts branching morphogenesis. Our findings highlight macrophages as novel therapeutic targets. Modulation of macrophage recruitment or polarization, as well as pharmacologic inhibition of downstream NF-κB signaling (e.g., with dexamethasone or selective inhibitors), represent promising strategies to restore normal lung development. Given the poor outcomes of severe CDH cases under current treatments, immunomodulatory therapies could open an entirely new avenue to improve survival and long-term pulmonary function in infants with CDH.
